# Repurposing of a human antibody-based microarray to explore conserved components of the signalome of the parasitic nematode *Haemonchus contortus*

**DOI:** 10.1186/s13071-022-05400-w

**Published:** 2022-07-30

**Authors:** Jack Adderley, Tao Wang, Guangxu Ma, Yuanting Zheng, Neil D. Young, Christian Doerig, Robin B. Gasser

**Affiliations:** 1 School of Health and Biomedical Sciences, RMIT University, Bundoora, VIC Australia; 2 Department of Veterinary Biosciences, Melbourne Veterinary School, The University of Melbourne, Parkville, VIC Australia; 3 College of Animal Sciences, Zhejiang Provincial Key Laboratory of Preventive Veterinary Medicine, Zhejiang University, Hangzhou, China; 4 Burnet Institute, Melbourne, VIC Australia

**Keywords:** Signalling, Antibody microarray, Repurposing antibodies, Kinases, Phosphorylation

## Abstract

**Background:**

Gaining insight into molecular signalling pathways of socioeconomically important parasitic nematodes has implications for understanding their molecular biology and for developing novel anthelmintic interventions.

**Methods:**

Here, we evaluated the use of a human antibody-based microarray to explore conserved elements of the signalome in the barber’s pole worm *Haemonchus contortus*. To do this, we prepared extracts from mixed-sex (female and male) adult worms and third-stage larvae (L3s), incubated these extracts on the antibody microarray and then measured the amounts of antibody-bound proteins (‘signal intensity’).

**Results:**

In total, 878 signals were classified into two distinct categories: signals that were higher for adults than for larvae of *H. contortus* (*n *= 376), and signals that were higher for larvae than for adults of this species (*n* = 502). Following a data-filtering step, high confidence (‘specific’) signals were obtained for subsequent analyses. In total, 39 pan-specific signals (linked to antibodies that recognise target proteins irrespective of their phosphorylation status) and 65 phosphorylation-specific signals were higher in the adult stage, and 82 pan-specific signals and 183 phosphorylation-specific signals were higher in L3s. Thus, notably more signals were higher in L3s than in the adult worms. Using publicly available information, we then inferred *H. contortus* proteins that were detected (with high confidence) by specific antibodies directed against human homologues, and revealed relatively high structural conservation between the two species, with some variability for select proteins. We also in silico-matched 763 compound structures (listed in the DrugBank and Kinase SARfari public databases) to four *H. contortus* proteins (designated HCON_00005760, HCON_00079680, HCON_00013590 and HCON_00105100).

**Conclusions:**

We conclude that the present antibody-based microarray provides a useful tool for comparative analyses of signalling pathways between/among developmental stages and/or species, as well as opportunities to explore nematocidal target candidates in *H. contortus* and related parasites.

**Graphical Abstract:**

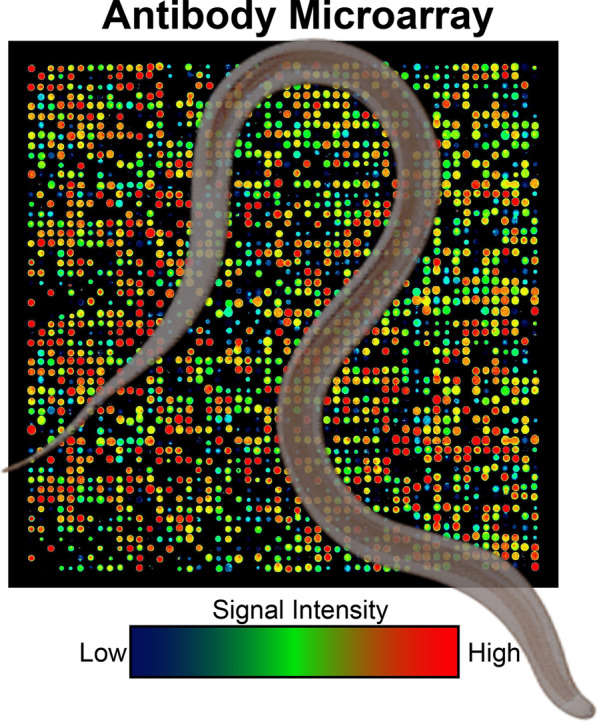

**Supplementary Information:**

The online version contains supplementary material available at 10.1186/s13071-022-05400-w.

## Background

The availability of genomes, transcriptomes and proteomes for the barber’ s pole worm (*Haemonchus contortus*) [1–5]—one of the most pathogenic nematodes of ruminants worldwide [6]—provides a solid foundation for detailed explorations of molecular pathways and processes in this nematode.* Haemonchus contortus* is representative of many species of order Strongylida, a large order of socioeconomically important parasitic roundworms (nematodes). Of particular significance in this context are signalling pathways, because of their crucial roles in a wide range of physiological and developmental processes. Many pathways are regulated by protein kinases, which are enzymes (transferases) that phosphorylate a substrate by transferring a phosphoryl group from an energy-rich molecule, such as adenosine triphosphate (ATP), to a target protein [7]. These kinases are classified into nine key groups and numerous families/subfamilies based on sequence similarity in their catalytic domains and the presence of accessory domains (cf. [8]). Despite the elevation of *H. contortus* to a ‘model organism’, much work remains to be done to create critical resources and reagents, such as antibody and nucleic acid probes, to identify and characterise key components of signalling pathways and processes.

The lack of commercially available antibodies against proteins from organisms that are not used as classical research models (e.g. mammalian cells, yeast, *Drosophila* and *Caenorhabditis*) is a hindrance to research. Consequently, research groups in smaller fields are often forced to generate their own antibodies to study particular proteins of interest, and this precludes system-wide studies involving large numbers of antibodies. In stark contrast, antibodies against human proteins, including those involved in phosphorylation-based signalling, are widely available, providing research with rapid and cost-effective tools. In recent years, a number of microarrays containing more than 2000 antibodies against various human proteins have reached the market [9]. One of these microarrays, the KAM series produced by Kinexus Bioinformatics Corp. (Vancouver, BC, Canada), contains antibodies against components of the human intracellular signalling environment, with an overall focus on phospho-signalling. This microarray has been employed by our team to identify signalling changes in the host cell during intracellular infection with a variety of viral, bacterial and protistan pathogens and endosymbionts [10–12]. Surprisingly, one of these studies showed that conservation in some signalling proteins between mosquito and human was sufficient to identify signalling changes in mosquito cells during infection with the bacterial endosymbiont *Wolbachia* [10]. This study highlighted the high level of conservation between key signalling proteins shared between evolutionarily distant metazoan taxa, particularly with respect to regulatory phosphorylation sites that are recognised by phospho-specific antibodies. In the study reported here, we employed a similar antibody microarray to: (i) identify antibodies that could be utilised to study homologous proteins in the nematode worm *H. contortus* and (ii) delineate differences in expression and phosphorylation of these proteins between the adult stage and third-stage larvae (L3s).

## Methods

### Procurement of *H. contortus*

*Haemonchus contortus* (Haecon-5 strain) was maintained in experimental sheep [13], in accordance with institutional animal ethics guidelines (approval permit no. 1714374; The University of Melbourne). Helminth-free Merino sheep (males; 8 months of age) were inoculated orally with 7000 L3s of *H. contortus*. Faecal samples containing *H. contortus* eggs were collected every day from 21 days following inoculation. These samples were incubated at 27 °C for 1 week to produce L3s [14]. Larvae were then harvested, sieved through two layers of nylon mesh (pore size: 20 μm; Rowe Scientific PTY, Ltd., Minto, NSW, Australia) to remove debris and dead larvae, washed 5 times in H_2_0 (volume), pelleted by centrifugation at 600 *g* (5 min) and, following removal of supernatant, frozen at − 80 °C. Adult worms (female and male) were collected from the abomasum of an infected sheep 28 days following inoculation with L3s, washed 5 times in 50-ml volumes of physiological saline, pelleted and then frozen at − 80 °C until use.

### Kinexus antibody microarray analysis

The KAM 900P antibody microarray kit (Kinexus Bioinformatics Corp.) was utilised. The KAM-900P chip uses 878 distinct antibodies to protein kinases and other cell signalling proteins; of these antibodies, 265 are pan-specific and 613 are phosphosite-specific. The antibodies are positioned on a Nexterion-P 3D matrix-coated glass slide, with duplicate spots in two distinct chambers to accommodate two separate samples. Signal detection is performed using a single, non-competitive dye. Cryopreserved *H. contortus* adults (male and female, in an approx. 50/50 mix) and cryopreserved L3s were separately subjected to freeze–thawing cycling in liquid nitrogen and homogenised in physiological saline (pH 7.4). The resultant cellular debris was removed from each sample by centrifugation (10,000 *g*), and proteins were extracted according to the manufacturer’s instructions to ensure that protease and phosphatase activity was minimised. The samples were loaded onto the microarray chambers for analysis using equal protein concentrations (2 mg/ml; 100 µg of protein for each sample). Following sample loading onto the microarray, the scanning and preliminary readout of the microarray were conducted by the Kinexus Bioinformatics Corp.

Microarray signals that were defined as “low intensity” as well as those with “high error” were removed from this analysis because they represented unreliable leads. Low-intensity signals were defined as signals where the globally normalised L3 signal and the globally normalised adult signal were both < 1000 units, consistent with the manufacturer’s recommendations for this antibody-based microarray system. High error signals were defined as signal differences, for which the percentage error of the L3 signals technical duplicates + the percentage signal error for the adult technical duplicates was greater than the magnitude of the percentage difference between the two developmental stages used in this study.

### Structure modelling and comparison of kinases and in silico-matching to chemical compounds in public data bases

The program AlphaFold (v2.0) [15] was used to predict the three-dimensional structures of proteins inferred to have differential signals to L3 or the adult stage of *H. contortus* in the antibody-based microarray. Proteins > 2500 residues in length were excluded due to technical limitations (relating to the central processing unit [CPU], graphic rocessing units [GPU] and/or random access memory [RAM]). The program TM-align [16] was used to structurally align homologous sequences between *H. contortus* and human (in a pairwise manner); a structural similarity was expressed as a TM-score, with a score of > 0.5 indicating that two structures are similar and related, and a score of < 0.2 indicating that they are unrelated. Structures were compared and displayed using the program UCSF ChimeraX [17]. The phosphosites and/or structural alignment of *H. contortus* orthologues were studied and displayed using the PyMOL molecular graphics system v2.5 [18].

The molecules for which antibody-based microarray signals were (1) higher in the *H. contortus* adult than in the L3s and (ii) matched a human homologue, were matched to sequences in the Kinase SARfari [19] and DrugBank v.4.3 [20] databases using PSI-BLAST v.2.2.26+, employing an E-value cut-off of 10^−30^ [8]. Chemicals in the Kinase SARfari database were considered if they met the rule-of-five [21] and were predicted to be “medicinal chemistry-friendly”.

## Results

To determine which human antibodies available in the KAM900 antibody microarray bound to *H. contortus* proteins and whether adult worms and larvae displayed different sets of signals, we prepared samples of mixed-sex (male and female) adult worms and L3 separately, and incubated the samples on the antibody microarray (one sample per chamber; see Methods section for details). The resultant 878 signals were sorted into two categories: signals that were higher in adults than in larvae (*n* = 376), and signals that were higher in larvae than in adults (*n* = 502). To establish which signals were reliable and warranted further investigation, two signal filtering steps were undertaken for the dataset; these removed signals with either low intensity or high error (see Methods section for details). The signal filtering step reduced the number of signals for further analyses to 39 pan-specific signals (linked to antibodies that recognise their target proteins irrespective of their phosphorylation status), 65 phosphorylation-specific signals which were higher in adult *H. contortus*, and 82 pan-specific signals and 183 phosphorylation-specific signals which were higher in the L3s (Table 1; “Total” vs. “Reliable”). A complete table of all signals measured is available (Additional file 1: Table S1; Additional file 2: Table S2).

**Table 1:** Summary of results for the antibody microarray signal filtering and lead signal determination for protein samples from adult and third-stage larvae of *Haemonchus contortus*

Microarray signals	Signals higher in adults	Signals higher in L3s
Total pan-specific	114	151
Low intensity	34	12
High error	41	57
Reliable	39	82
Total phospho-specific	262	351
Low intensity	89	51
High error	108	117
Reliable	65	183

*L3s* Third-stage larvae

**Fig. 1:**
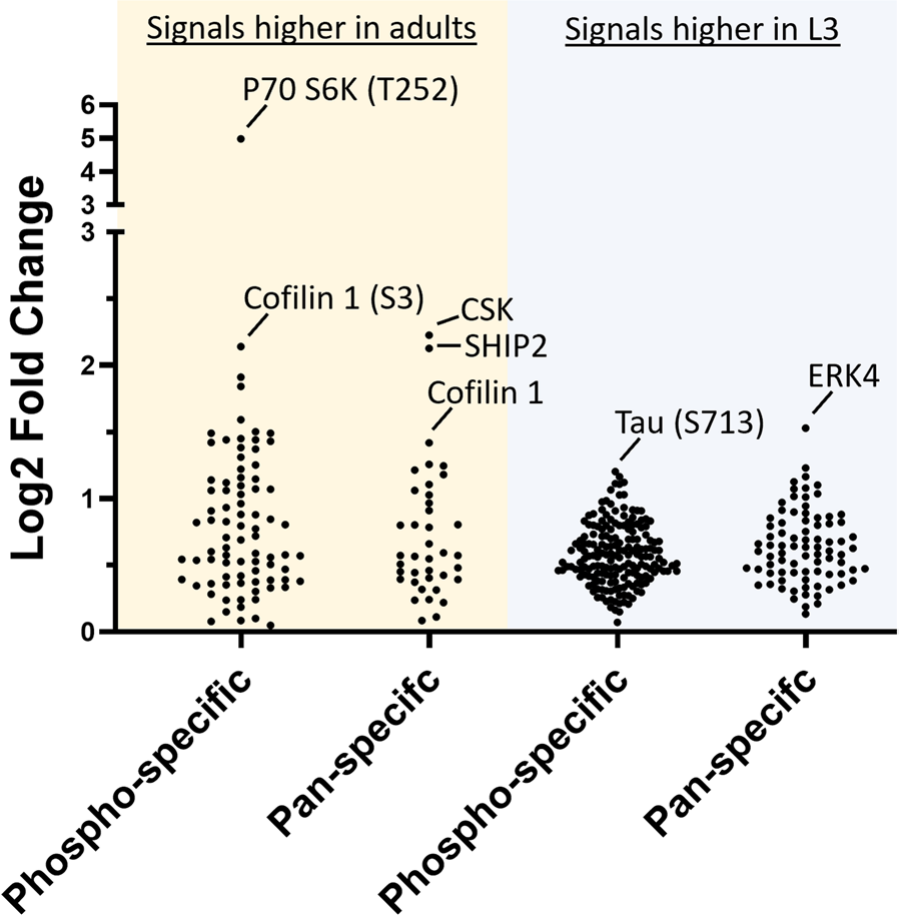
Dot plot of antibody microarray results separated according to which signals were higher in the adult stage or L3 of *Haemonchus contortus*. Signals represent the log2-fold change between the adult and L3 samples compared. Left-side of dot plot: signals for the pan-specific and phosphorylation-specific antibodies on the array that were higher in the adult stage than in L3. Righ-side of dot plot: signals for the pan-specific and phosphorylation-specific antibodies on the array that were higher in L3 than in the adult stage. The largest log2-fold change-associated signals are annotated above each plot. Signals which were flagged as low intensity and affected by high error have not been included here (signal filter strategy used is described in the Methods section). L3, Third-stage larvae; also see Abbreviation list

The signals classified as reliable were explored further. To aid visual interpretation of log2-fold change between the two development stages of *H. contortus*, we represented the signal differences between the samples in a scatter plot (Fig. 1). For each of the comparisons conducted here, there were several notable signals based on the magnitude of their fold changes, including those linked to the molecules ribosomal protein S6 kinase beta-1 (pP70 S6K), Cofilin 1, cytosolic tyrosine kinase (CSK), SHIP2 (SH2-containing phosphatidylinositol 3,4,5-trisphosphate 5-phosphatase) and ERK4 (mitogen-activated extracellular signal-regulated kinase 4). Following signal filtering, there were notably more signals that were higher in L3s than in the adult stage (2-fold more for pan-specific signals, 3-fold more for phospho-specific signals). This finding is consistent with the increased signalling activity expected in developing (vs. terminally differentiated) tissues. Importantly, we identified which antibodies were highly likely to recognise a genuine *H. contortus* homologue of the human target protein. This was made possible through the availability of the antibody epitopes and uniport ID for each human protein targeted on the microarray. Using publicly available genomic and transcriptomic data sets from Parasite WormBase [2, 22], we identified nucleotide sequences coding for the proteins represented by high signals in the microarray (Table 2). Manual curation of the protein sequences identified distinct homologues between human and *H. contortus* (Additional file 3: Figure S1). Only protein sequence matches with an identity/E-value of 10^–5^ and fold change of > 2.0 were included. The complete, matched list of antibody signals to the *H. contortus* proteins is provided in Table 1. Subsequent comparative modelling of these proteins revealed, overall, relatively high structural conservation between the two species, although some sub-structural elements within some proteins (e.g. HCON_00125210 and HCON_00080620; Additional file 3: Fig. S1) were variable (< 50% on colour scale). In silico-matching to chemicals in public databases (i.e. DrugBank and Kinase SARfari databases) revealed 763 compounds that matched four *H. contortus* proteins (HCON_00005760, HCON_00079680, HCON_00013590 and HCON_00105100; Table 3; Additional file 4: Table S3; Additional file 5: Table S4).

**Table 2:** Summary of *H. contortus* proteins for which antibody-based microarray signals were high in the adult (*n* = 18) versus the third-stage larvae (*n* = 17) and had a matched human homologue

Human target protein	Phospho-site (Human)	Phosphosite sequence	Uniprot ID	Fold-change	*H. contortus* homologue	TM-score	Conserved phospho-site^a^	Stage
p70 S6K	T252	TL(pS)PI	P23443	31.62	HCON_00079680	0.71201	Yes	Adult
ELK1	S383	MA(pS)GVA	P19419	2.61	HCON_00127560	0.3449	No	Adult
eIF4E	S209	KSG(pS)TTK	P06730	2.38	HCON_00133690	0.81774	Yes	Adult
Integrin a4	S1021	RRD(pS)WSY	P13612	2.23	HCON_00080620	0.50131	Yes	Adult
Histone H3	S28	ARK(pS)APS	P84243	2.21	HCON_00065870	0.96784	Yes	Adult
Histone H3	T3	VTH(pT)FCG	P84243	2.17	HCON_00065870	0.96784	Yes	Adult
SYK	Y323	AR(pT)KQT	P43405	2.14	HCON_00013780	0.53671	No	Adult
Crystallin aB	S19	PFH(pS)PSR	P02511	2.10	HCON_00114930	0.49117	Yes	Adult
Histone H2B	S14	SAPAPKKG(pS)KK	P33778	2.09	HCON_00026210	0.74892	Yes	Adult
PI3K	Pan-specific	na*	P27986	4.68	HCON_00017820	0.33794	na	Adult
CSK	Pan-specific	na	P41240	4.37	HCON_00013590	0.69703	na	Adult
Arrestin b	Pan-specific	na	P49407	2.39	HCON_00167390	0.87674	na	Adult
Crystallin aB	Pan-specific	na	P02511	2.37	HCON_00114930	0.49117	na	Adult
Bmx	Pan-specific	na	P51813	2.32	HCON_00026740	0.47372	na	Adult
HSP90a/b	Pan-specific	na	P07900	2.27	HCON_00136990	0.92043	na	Adult
CDK7	Pan-specific	na	P50613	2.15	HCON_00005760	0.9175	na	Adult
CDK1	Pan-specific	na	P06493	2.08	HCON_00101500	0.95318	na	Adult
IkBa	Pan-specific	na	P25963	2.04	HCON_00125210	0.20125	na	Adult
SgK269	Y635	PNA(pY)DNL	Q9H792	2.30	HCON_00028850	0.23692	No	L3
YSK1	T174	KRN(pT)FVG	O00506	2.18	HCON_00162520	0.64192	Yes	L3
InsR (IR)	Y1189	ETD(pY)YRK	P06213	2.16	HCON_00066590	0.51222	Yes	L3
MAPKAPK2	Y225 + T226	PC(pY)(pT)PYYV	P49137	2.15	HCON_00101270	0.83651	Yes	L3
ErbB3 (HER3)	Y1307	HVH(pY)ARL	P21860	2.09	HCON_00018780	0.32582	No	L3
B-Raf (RafB)	S729	RSA(pS)EPS	P15056	2.04	HCON_00111880	0.41322	Yes	L3
VIM	Y117	FAN(pY)IDK	P08670	2.04	HCON_00059550	0.41927	Yes	L3
ERK4 (MAPK4)	Pan-specific	na	P31152	2.89	HCON_00091920	0.64358	na	L3
JAK2	Pan-specific	CWNNNVNQRPSFRDLA	O60674	2.34	HCON_00109060	0.29177	na	L3
MKK3	Pan-specific	CTDIAAFVKEILGEDS	P46734	2.24	HCON_00105100	0.84979	na	L3
ASK1	Pan-specific	na	Q99683	2.18	HCON_00040310	0.64569	na	L3
WNK1	Pan-specific	CLETKAVGMSNDGRFL	Q9H4A3	2.14	HCON_00107110	0.29247	na	L3
RET	Pan-specific	CKRPVFADISKDLEKM	P07949	2.11	HCON_00187950	0.3504	na	L3
B-Raf (RafB)	Pan-specific	CSDDWEIPDGQITVGQ	P15056	2.10	HCON_00111880	0.41322	na	L3
EphA1	Pan-specific	na	P21709	2.05	HCON_00042040	0.32568	na	L3
MKK3	Pan-specific	CAERMSYLELMEHPFF	P46734	2.02	HCON_00105100	0.84979	na	L3
VEGFR2 (KDR)	Pan-specific	CILQPDSGTTLSSPPV	P35968	2.01	HCON_00185500	0.26013	na	L3

*na* not available

^a^The structural conservation of phospho-sites is shown in Additional file 3: Figure S1

**Table 3:** Predicted kinase inhibitors that matched four *H. contortus* proteins representing two developmental stages—adult and third-stage larvae

Protein encoded by *Haemonchus contortus* gene	Developmental stage	Number of compounds in DrugBank database^a^	Number of compounds in Kinase SARfari database^a^
HCON_00005760	Adult	2	0
HCON_00079680	Adult	4	0
HCON_00013590	Adult	12	735
HCON_00105100	L3	10	0

^a^Individual compound codes are given in Additional file 4: Table S3; Additional file 5: Table S4, respectively

## Discussion

Gaining insight into molecular signalling pathways of socioeconomically important parasitic nematodes has major relevance for developing new interventions against the diseases that these worms cause in animals and humans [23–25], because it should be possible to identify targets in these pathways for the design of new anthelmintics. Given the major adverse impact of diseases caused by these worms, this focus is critical. Anthelmintic treatment is a key component of most parasite control programmes, but resistance to most available anthelmintic classes has become widespread in nematode populations around the world.

In addition to the benefits of employing this microarray-based assay as a tool for comparative studies of signalling pathways in pathogens, there is major merit in utilising information on essential gene products to predict and prioritise new anthelmintics to combat *H. contortus* and related nematodes (cf. [26]). At the present time, the small number of classes of anthelmintics available [27, 28] and the problem of widespread anthelmintic resistance [29] demand concerted efforts to discover new anthelmintic drugs with novel mechanisms/modes of action. Thus, some of the proteins identified here in the adult stage of *H. contortus* (which is the blood-feeding and pathogenic stage of the nematode) could be promising target candidates to evaluate, as the druggability of protein kinases is well-established [30], with > 70 kinase inhibitors on the market and > 150 in development.

The set of kinase inhibitors predicted here (*n* = 763), inferred to match three homologues in adults and in L3 of *H. contortus* (cf. Table 2), could be a useful starting point for targeted compound screening on *H. contortus*. Thus, a subset of these compounds could be selected as drugs, depending on the purchase cost, availability, chemical properties, safety and/or prior use(s), and tested for anthelmintic effects in a recently established, automated, whole-worm motility screening assay [15]. This step would be followed by a hit-to-lead phase, in which structural analogues would be synthesised and studied to establish structure–activity relationships, with the aim to maximise nematocidal effect and minimise binding to mammalian (host) homologues. Subsequent work could then focus on intestinal absorption, distribution, metabolism, excretion and toxicity assessments.

Given that *H. contortus* shares many orthologues with other clade V nematodes, the current work provides a starting point for the identification of novel drug targets in a range of socioeconomically important parasitic nematodes of human health importance (e.g. hookworms *Necator americanus* and *Ancylostoma duodenale*) and veterinary significance (e.g. species of *Trichostrongylus*, *Teladorsagia, Ostertagia*, *Cooperia* and many more). Moreover, the free-living nematode *Caenorhabditis elegans* (clade V), arguably the best characterised multicellular organism, could be used as a complementary or surrogate tool for the validation of targets and the mechanisms/modes of action of optimised compounds and for the prediction of resistance development in nematodes.

## Conclusion

In conclusion, the present antibody-based microarray has opened the door to fundamental investigations of signalling pathways in free-living and parasitic nematodes, and also between distinct developmental stages of these worms, and provides opportunities for the discovery and exploration of critical targets and new anthelmintics.

## Supplementary Information

**Additional file 1: Table S1.** Complete list of signals recorded as being higher in the adult stage of *Haemonchus contortus* than in the larval stage (L3)—in the Kinexus antibody microarray experiment.

**Additional file 2: Table S2. **Complete list of signals recorded as being higher in the larval stage (L3) of *Haemonchus contortus* than in the adult stage—in the Kinexus antibody microarray experiment.

**Additional file 3: Figure S1. **Three-dimensional structural models for *Haemonchus contortus* proteins with high antibody-based microarray signals, compared with their orthologues in *Homo sapiens* in a pairwise manner. Conserved regions are in pink, and divergent ones in green. The phosphosites in the protein sequence and structure is indicated in green and box, respectively.

**Additional file 4: Table S3. **Chemicals in the DrugBank database associated with the detected *Haemonchus contortus* proteins in the present study

**Additional file 5: Table S4. **Chemicals in KinaseSARfari database associated with the detected *Haemonchus contortus* proteins in the present study

## Data Availability

All data generated or analysed during this study are included in this article.

## Abbreviations

ATP: Adenosine triphosphate (ATP)
CSK: 50-KDa cytosolic tyrosine kinase
ERK4: Mitogen-activated extracellular signal-regulated kinase 4
L3s: Third-stage larvae
p70 S6K: Ribosomal protein S6 kinase beta-1
SHIP2: SH2-containing phosphatidylinositol 3,4,5-trisphosphate 5-phosphatase
