# Supplementary material for: Repurposing of a human antibody-based microarray to explore conserved components of the signalome of the parasitic nematode Haemonchus contortus
Source: Parasit Vectors. 2022 Jul 30;15:273. doi: 10.1186/s13071-022-05400-w (PMC9338626; doi:10.1186/s13071-022-05400-w)
Supplement: Supplementary file 3 — Additional file 3: Figure S1. Three-dimensional structural models for Haemonchus contortus proteins with high antibody-based microarray signals, compared with their orthologues in Homo sapiens in a pairwise manner. Conserved regions are in pink, and divergent ones in green. The phosphosites in the protein sequence and structure is indicated in green and box, respectively. [file 13071_2022_5400_MOESM3_ESM.docx]

**Figure S1**. Three-dimensional structural models for *Haemonchus contortus* proteins with high antibody-based microarray signals, compared with their orthologs in *Homo sapiens* in a pairwise manner. Conserved regions are in pink, and divergent ones in green. The phosphosites in the protein sequence and structure is indicated in green and box, respectively.

**
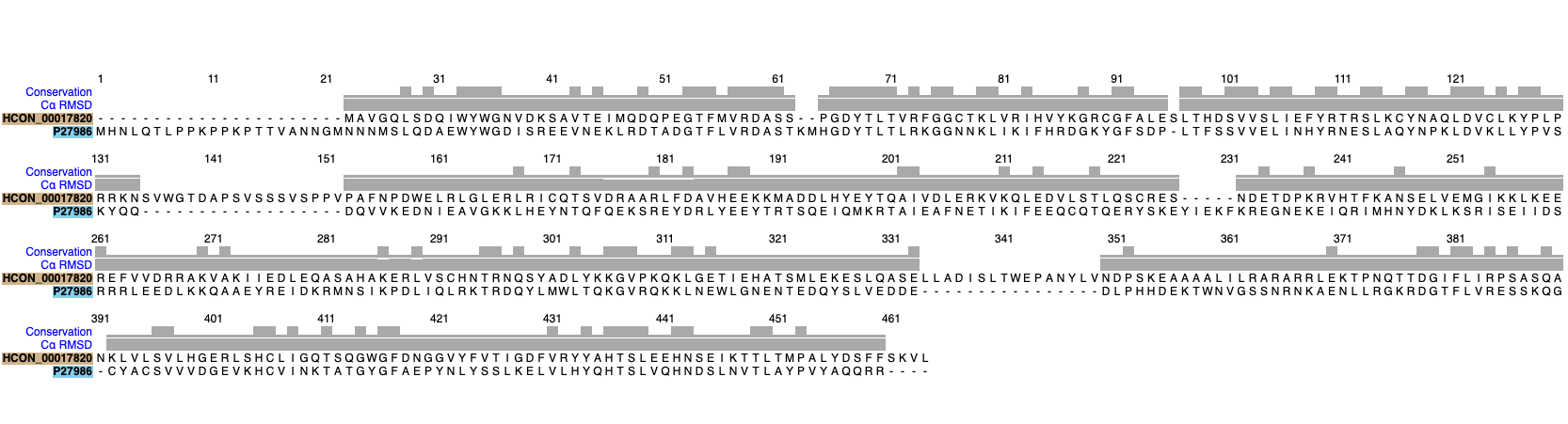
**

**
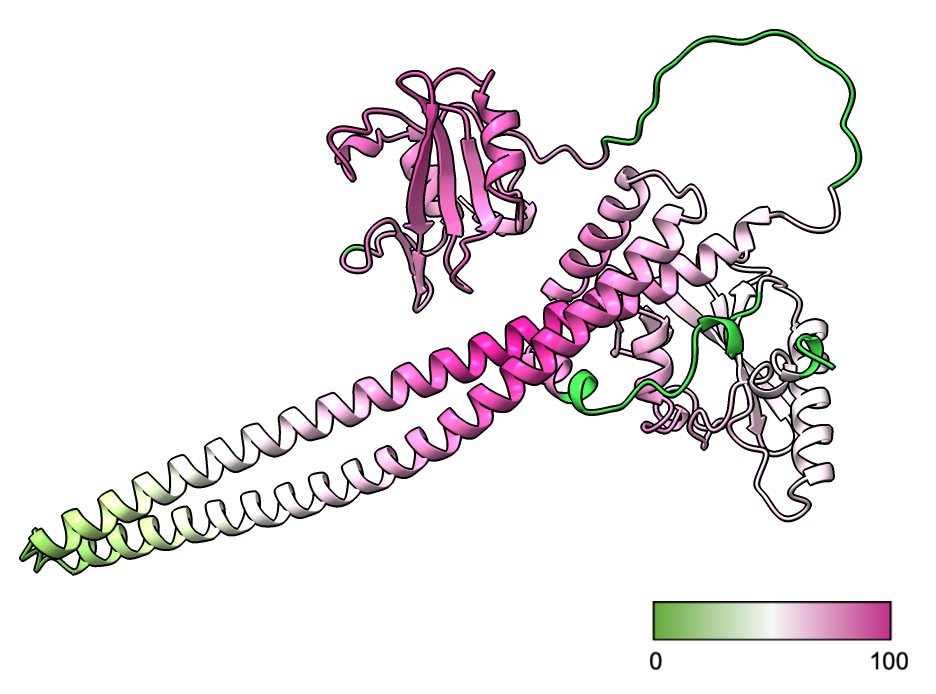
**

P27986 (*Homo sapiens*) vs HCON_00017820 (*Haemonchus contortus*)

**
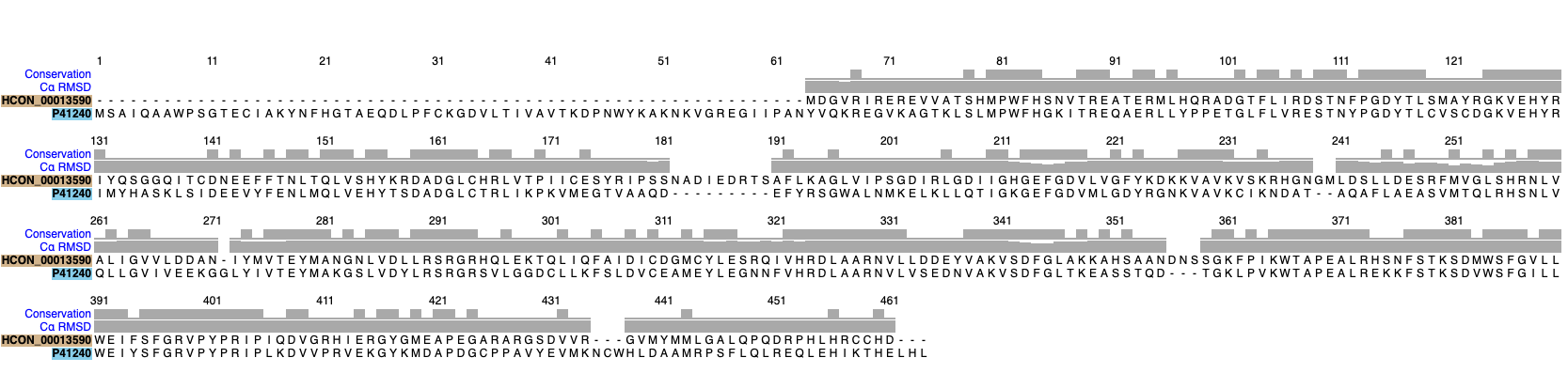
**

**
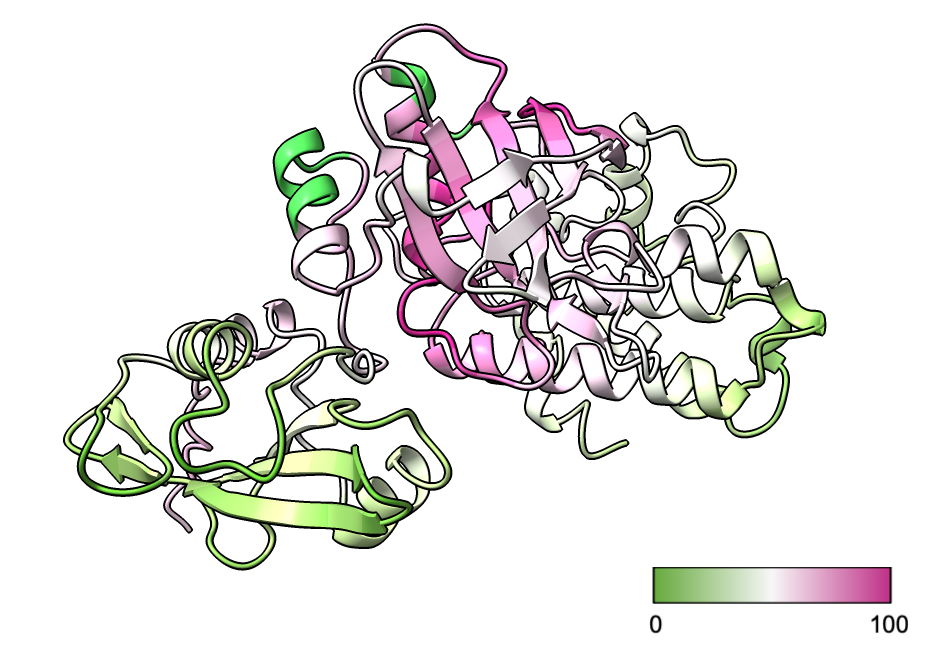
**

P41240 (*Homo sapiens*) vs HCON_00013590 (*Haemonchus contortus*)

**
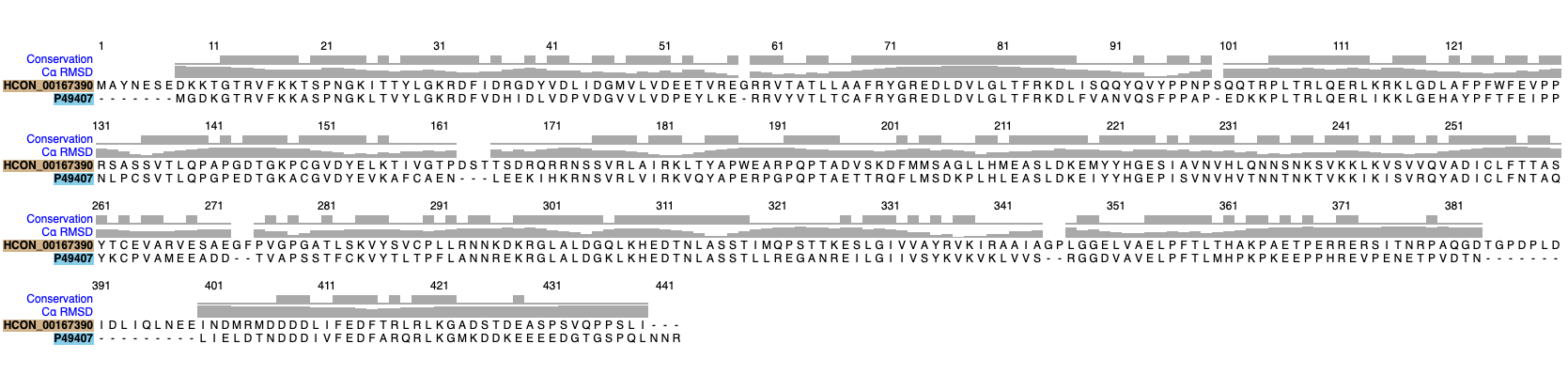
**

**
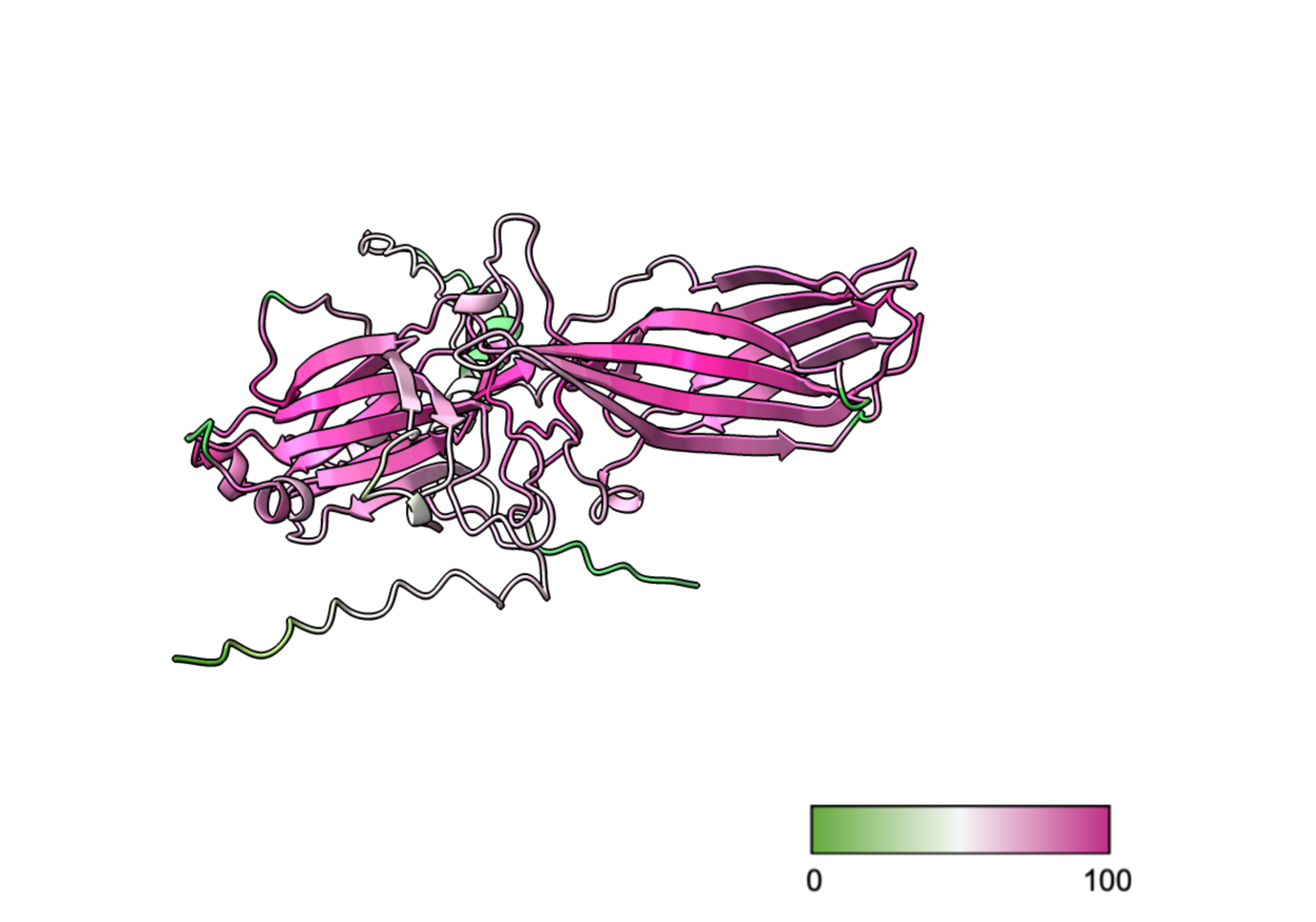
**

P49407 (*Homo sapiens*) vs HCON_00167390 (*Haemonchus contortus*)

**
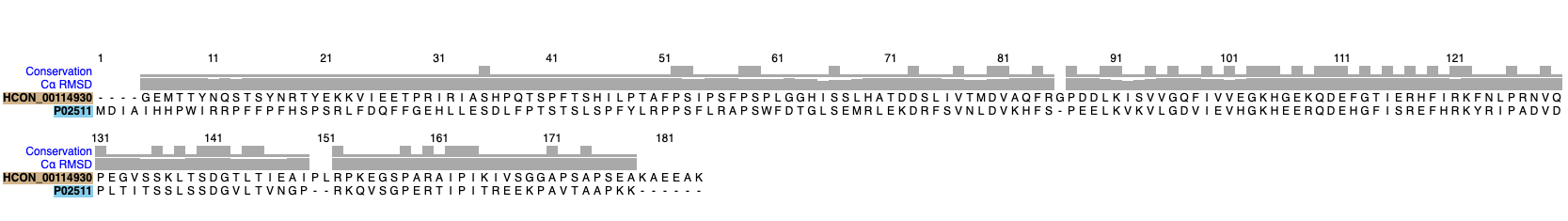
**

**
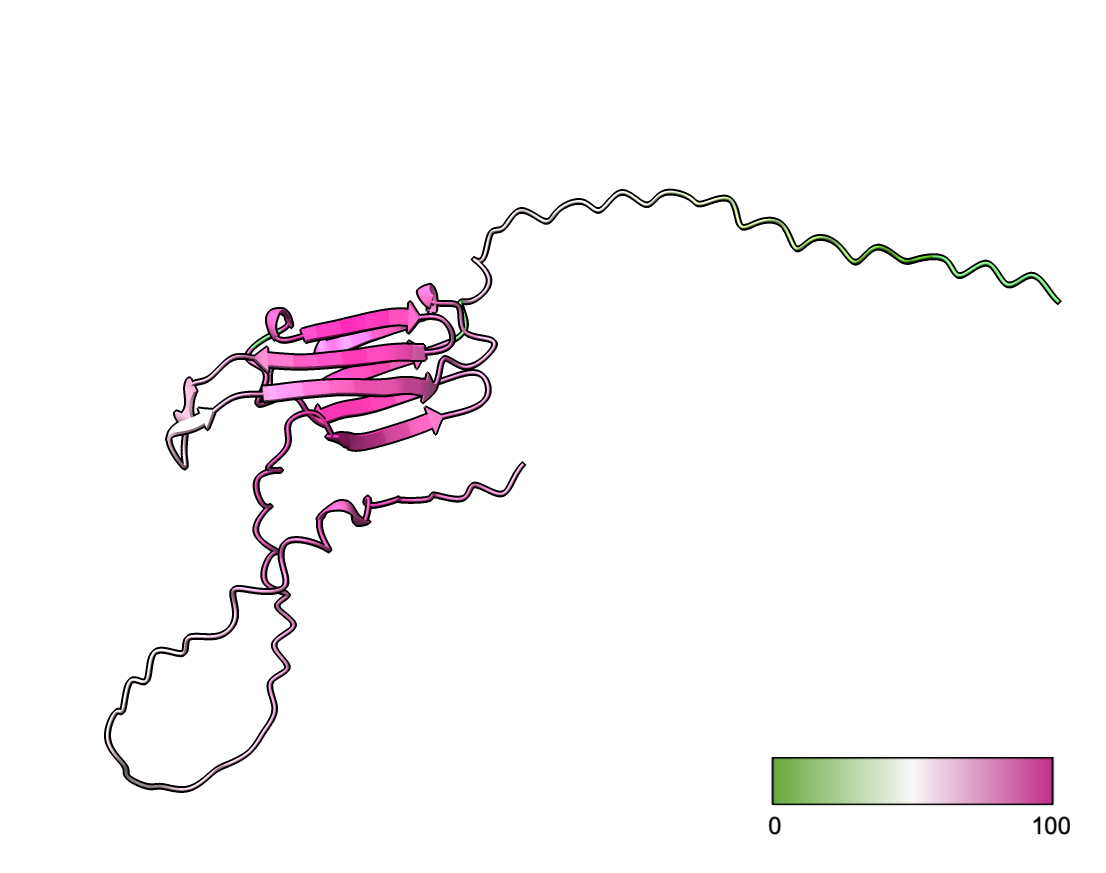
**

P02511 (*Homo sapiens*) vs HCON_00114930 (*Haemonchus contortus*)


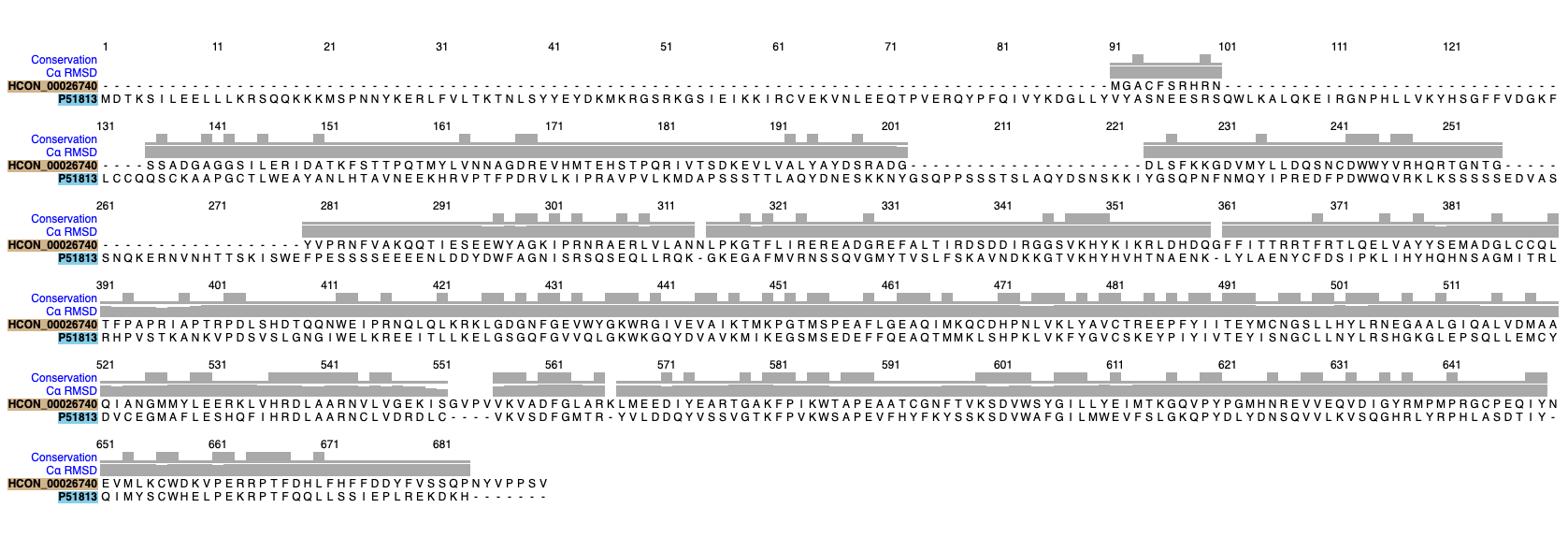


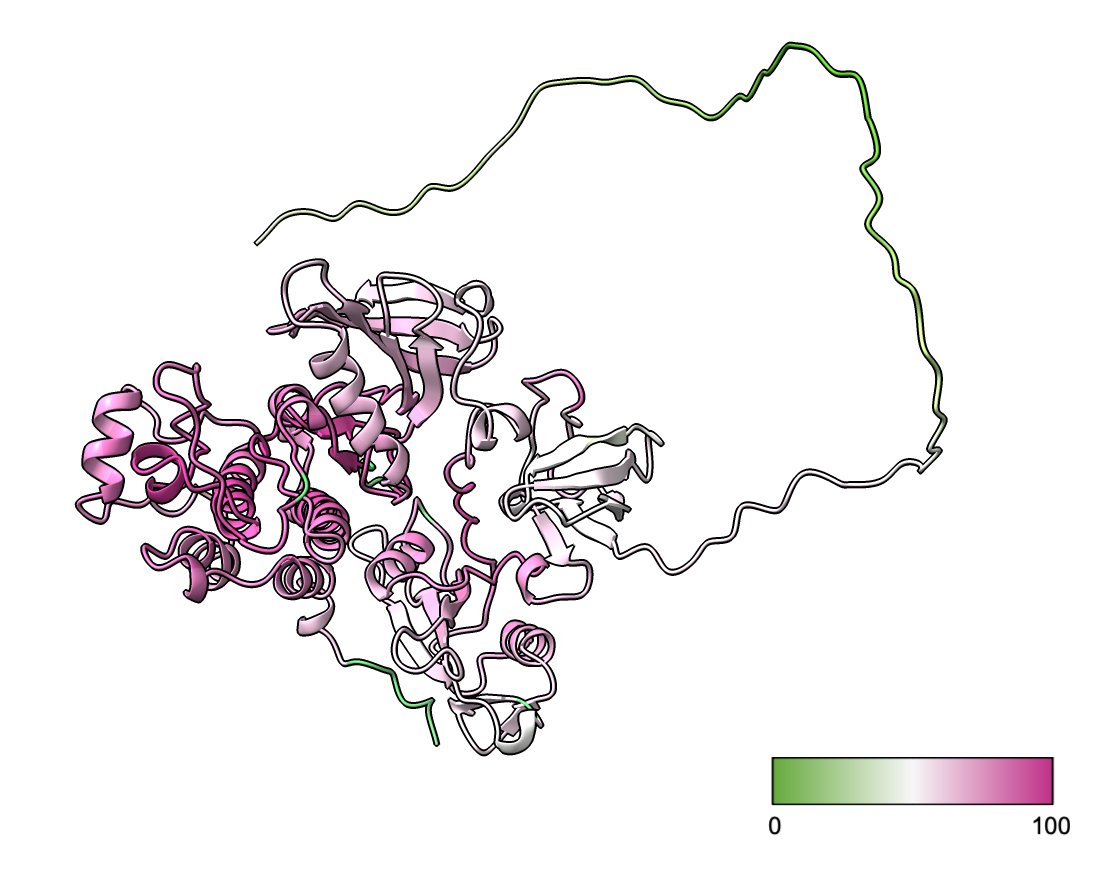


P51813 (*Homo sapiens*) vs HCON_00026740 (*Haemonchus contortus*)


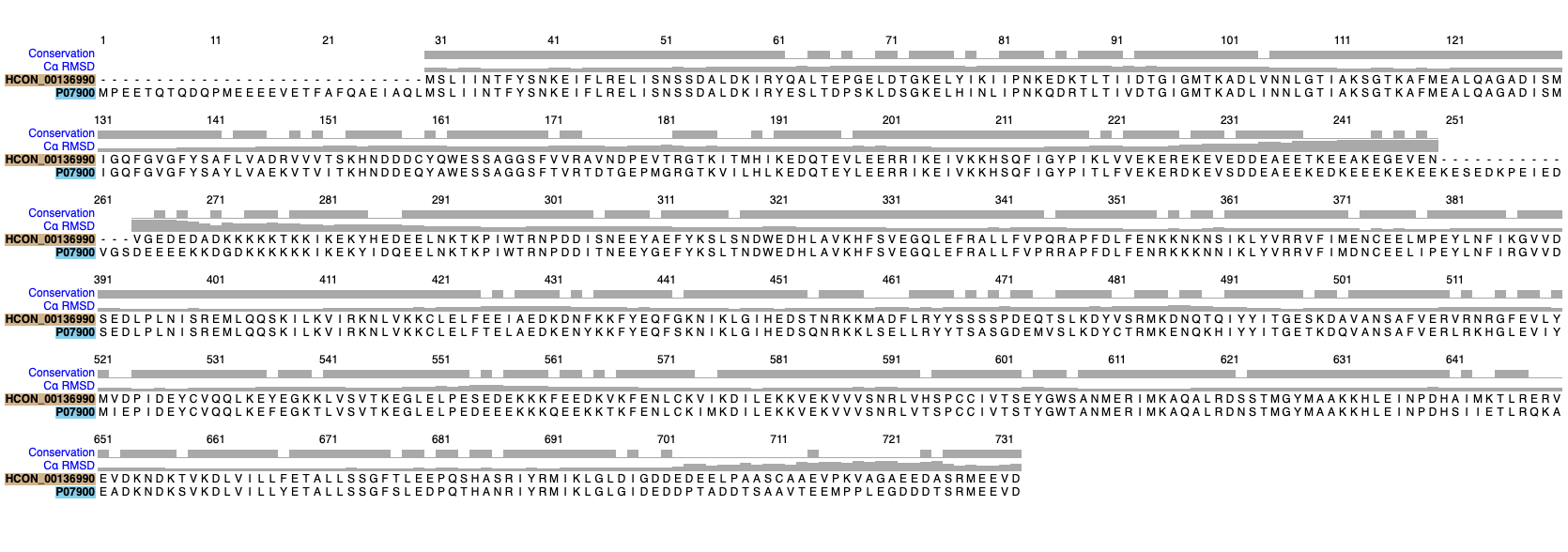

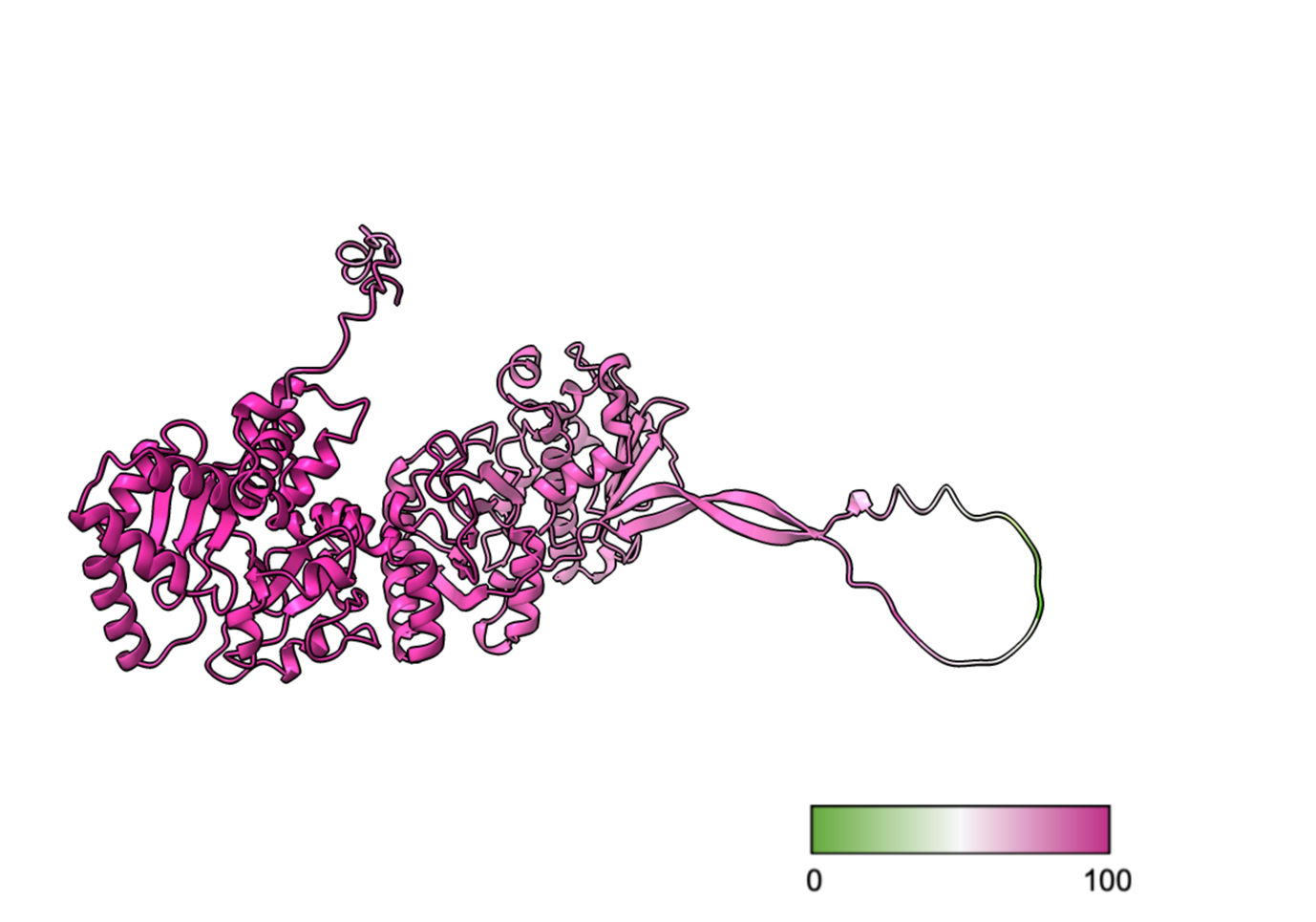


P07900 (*Homo sapiens*) vs HCON_00136990 (*Haemonchus contortus*)


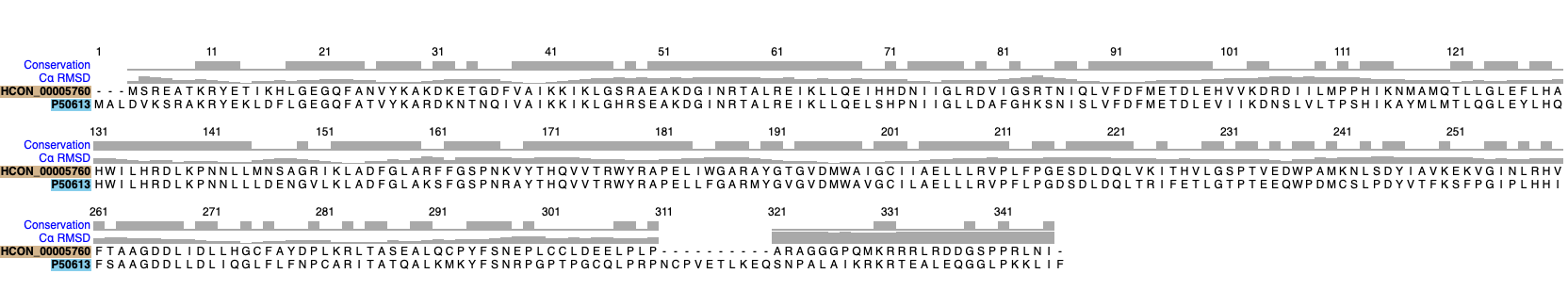


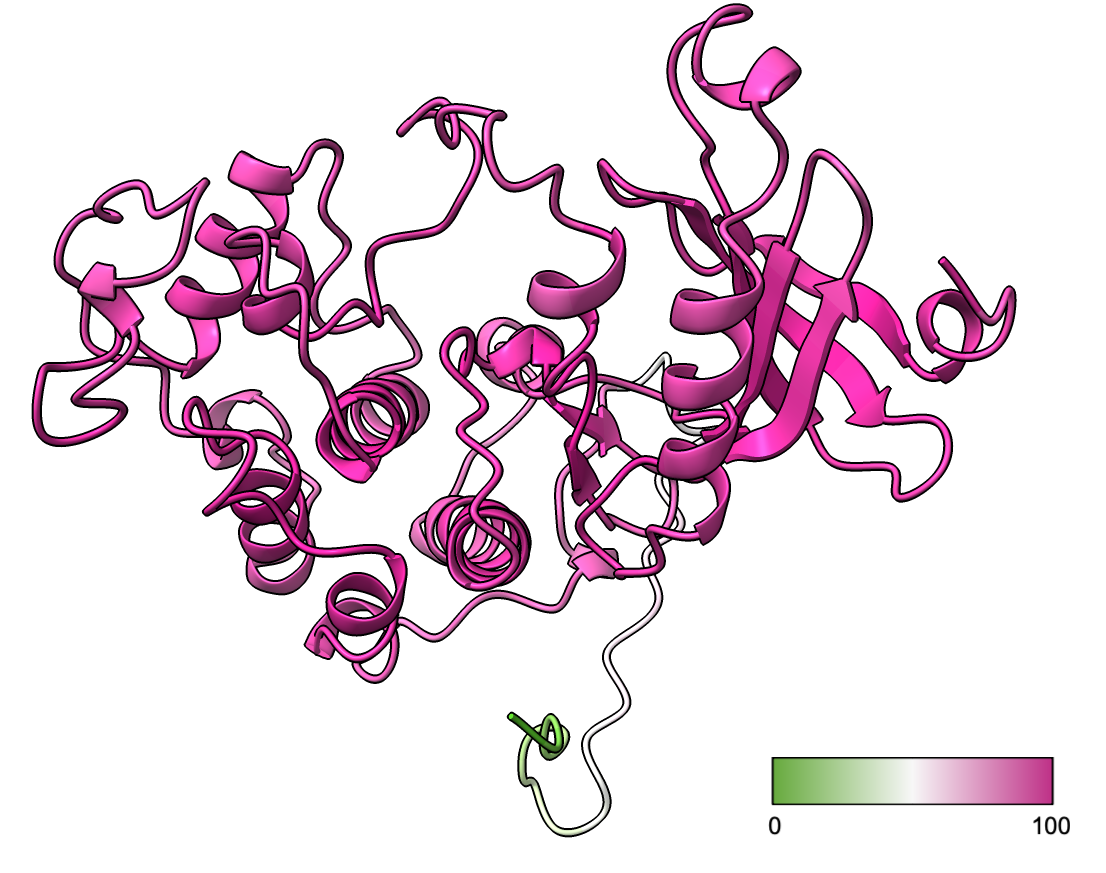


P50613 (*Homo sapiens*) vs HCON_00005760 (*Haemonchus contortus*)


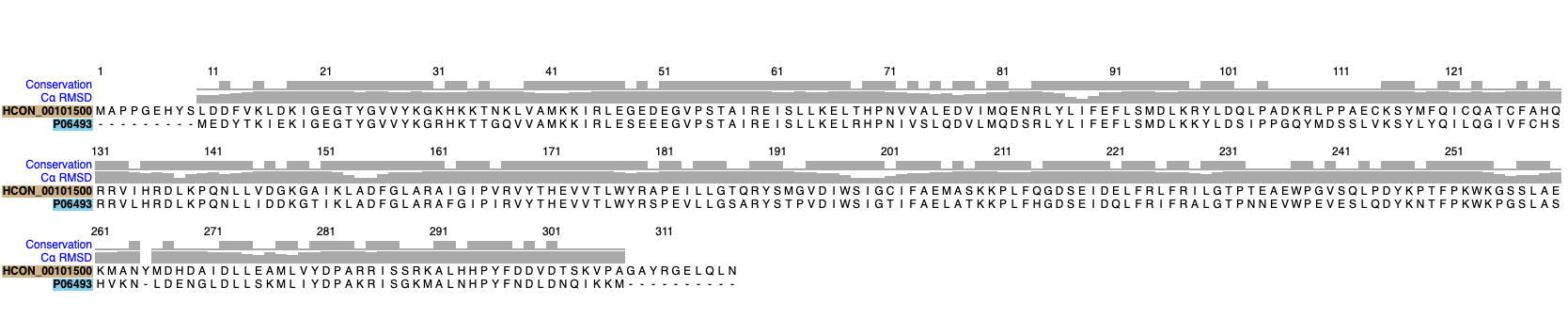

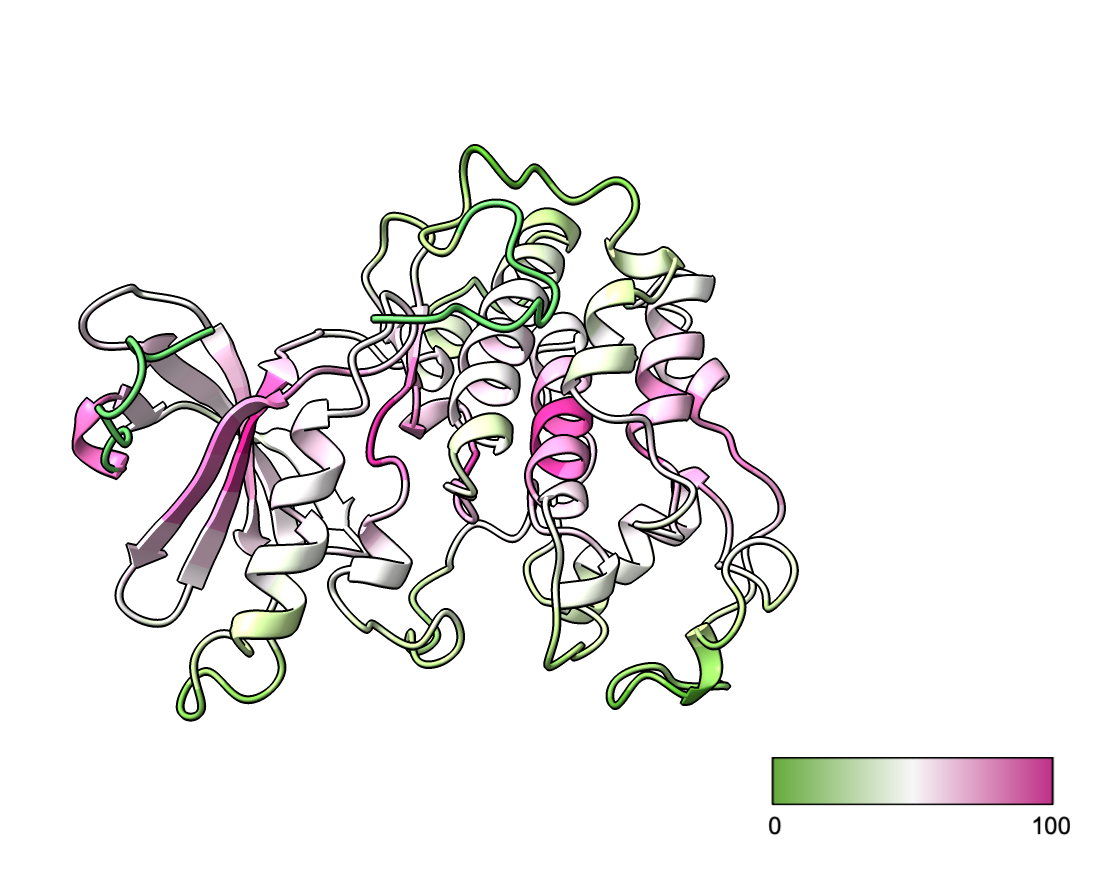


P06493 (*Homo sapiens*) vs HCON_00101500 (*Haemonchus contortus*)


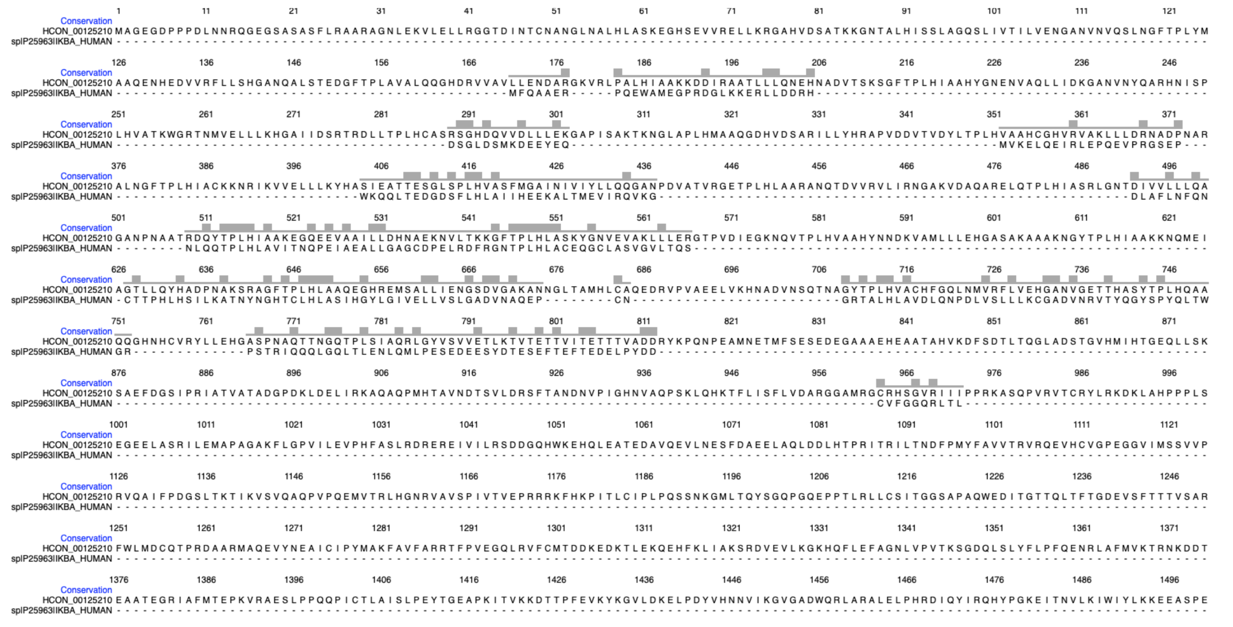


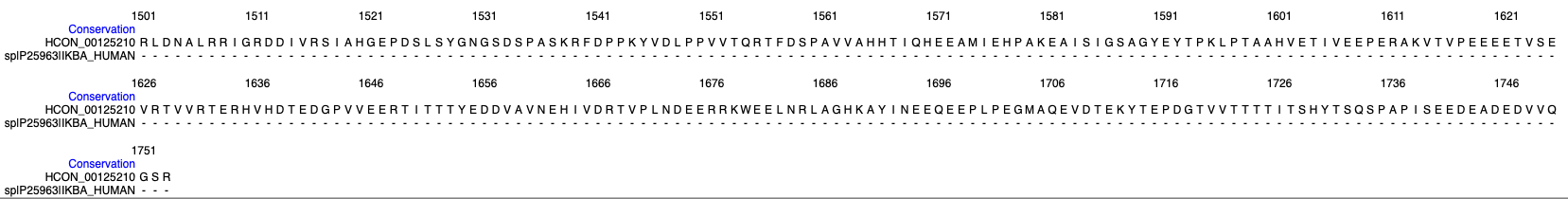


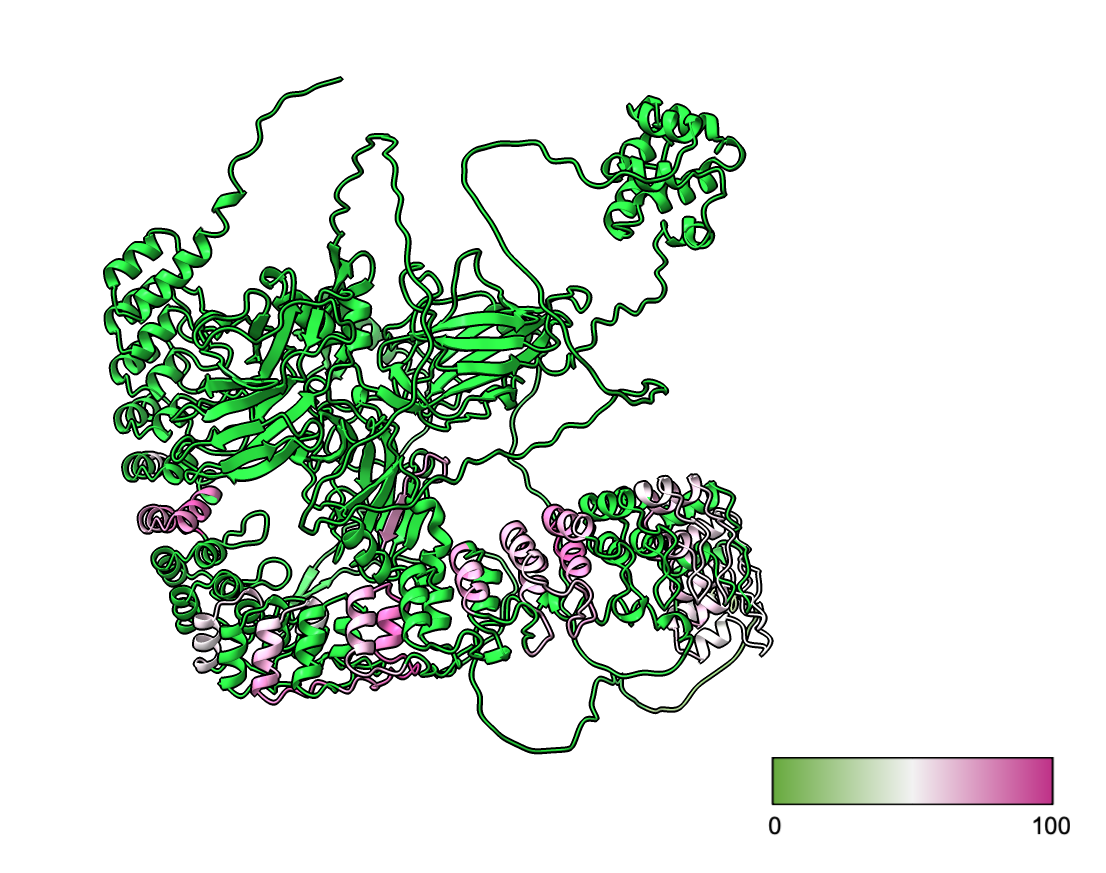


P25963 (*Homo sapiens*) vs HCON_00125210 (*Haemonchus contortus*)


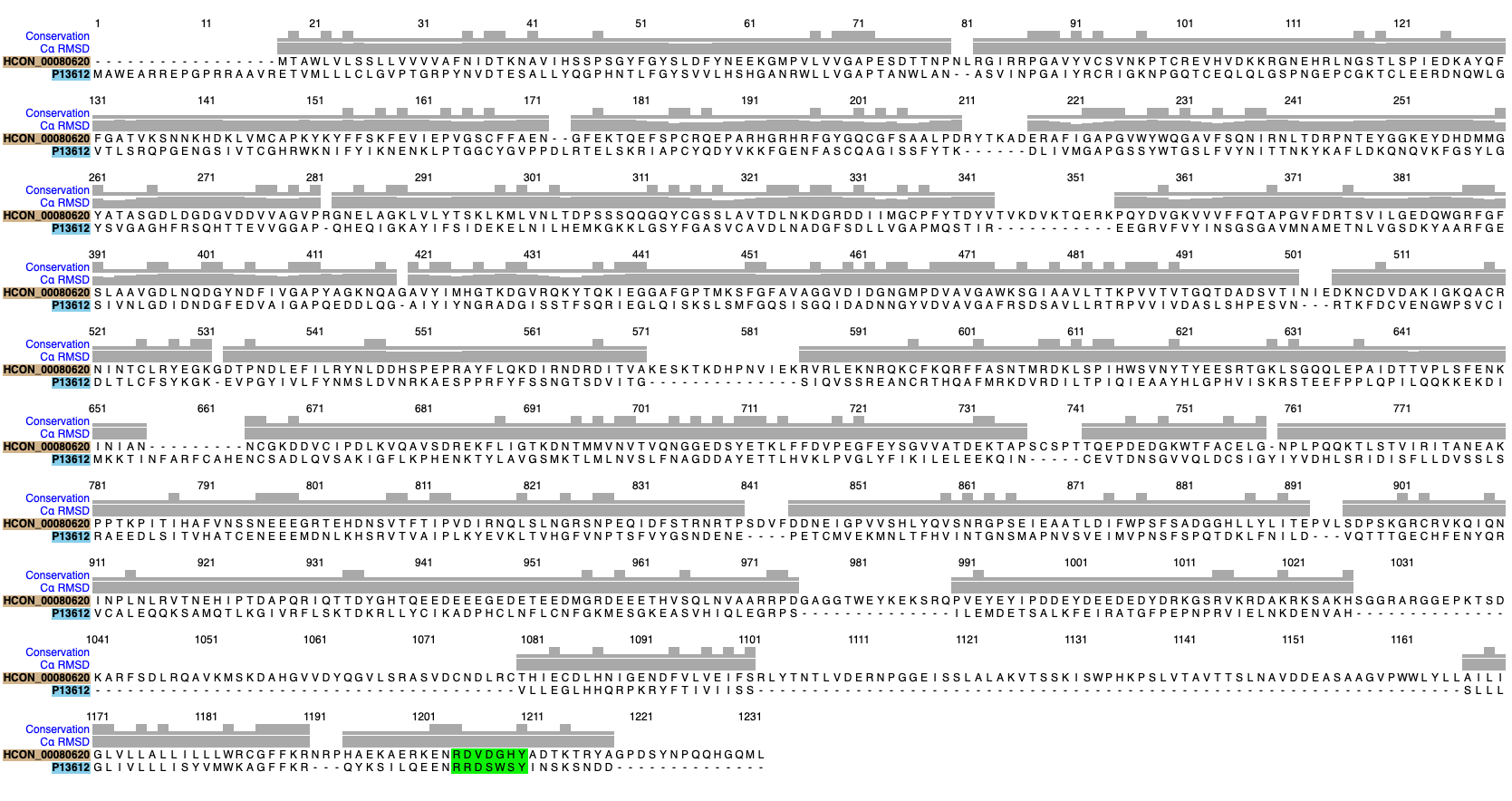


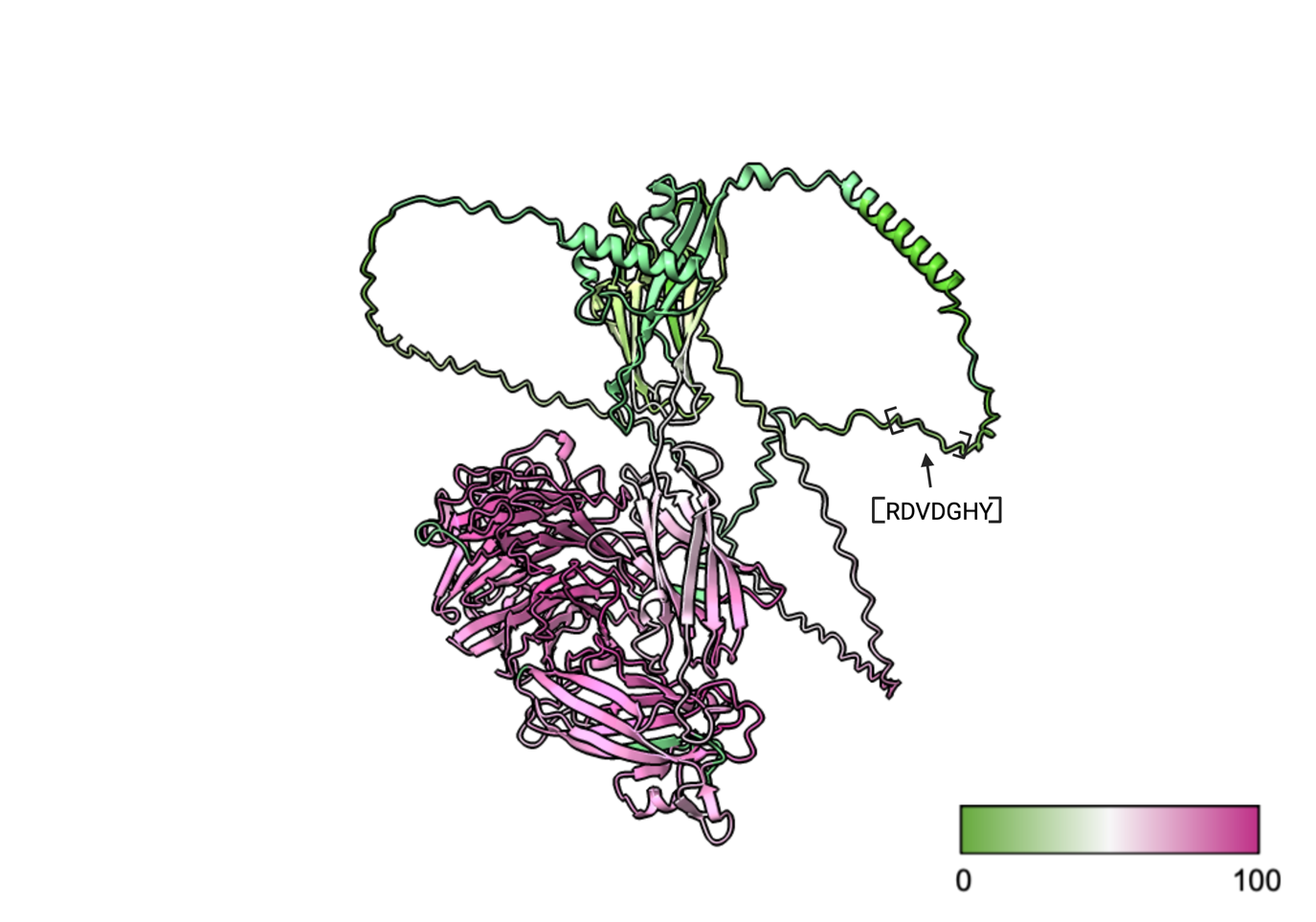


P13612 (*Homo sapiens*) vs HCON_00080620 (*Haemonchus contortus*)


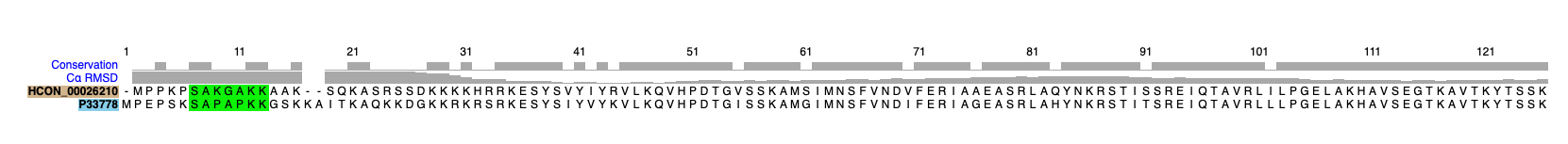

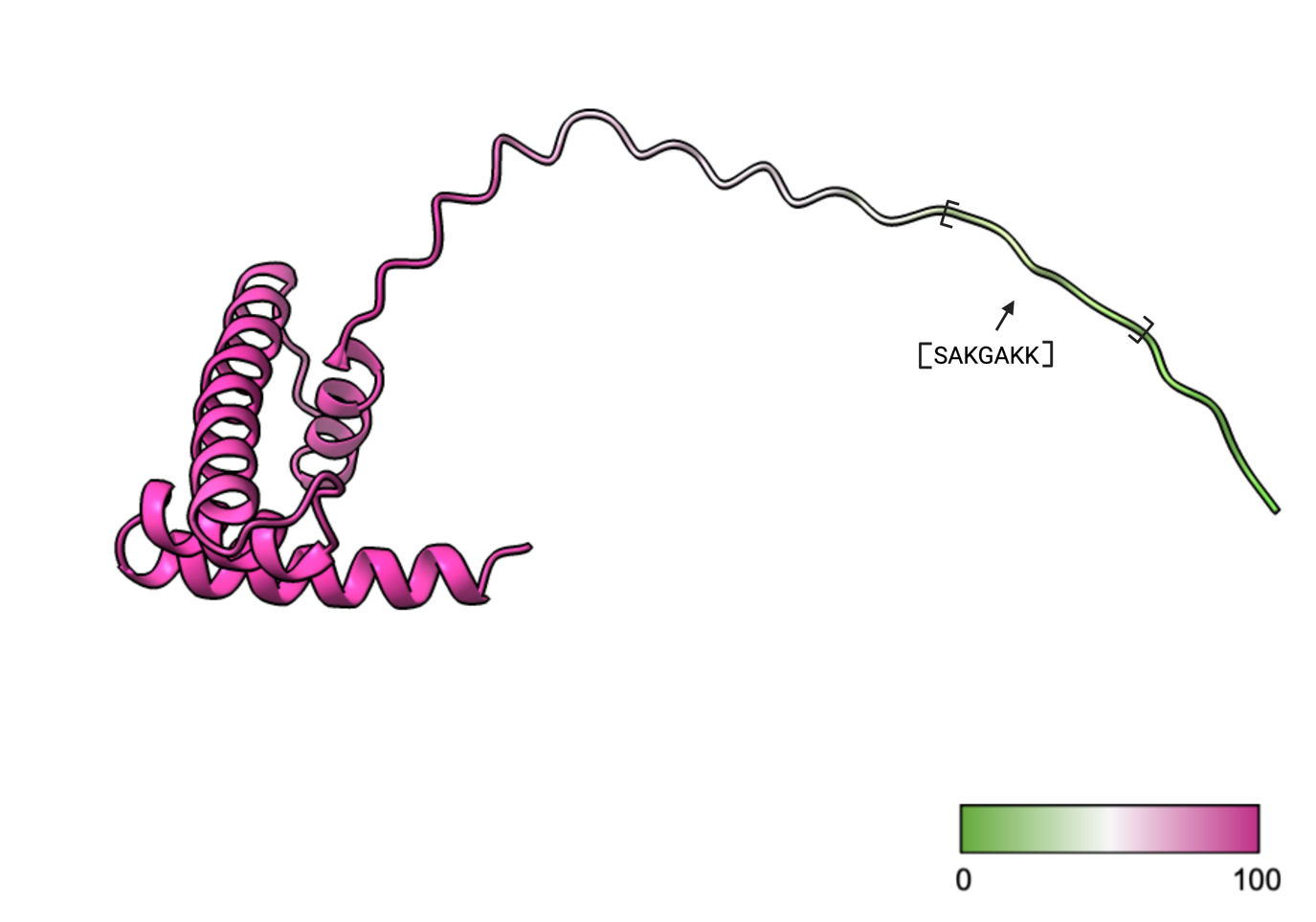
 P33778 (*Homo sapiens*) vs HCON_00026210 (*Haemonchus contortus*)


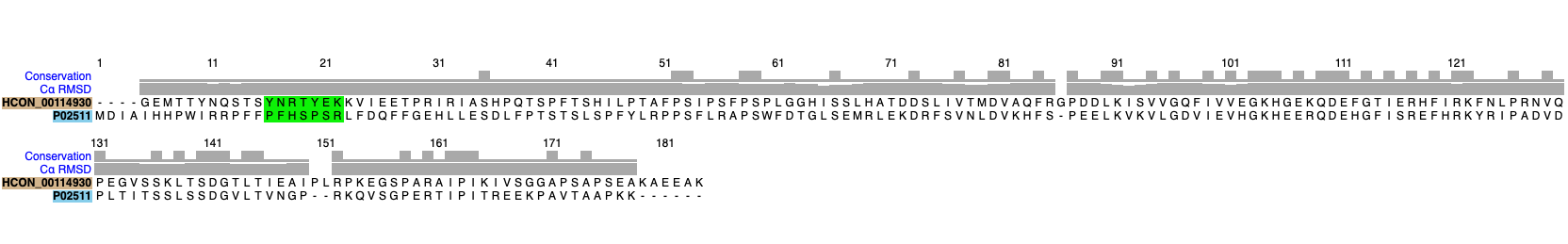

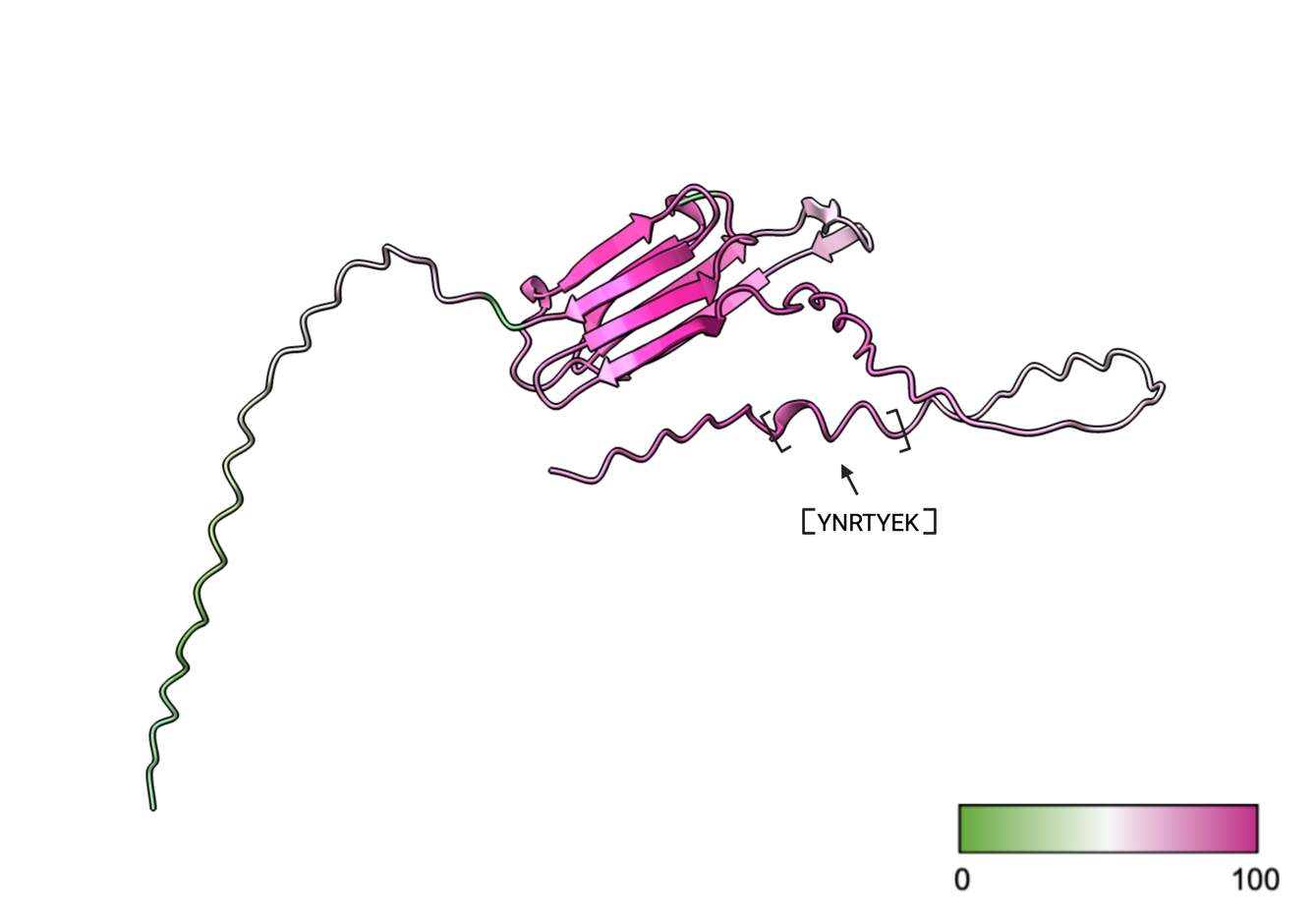
 P02511 (*Homo sapiens*) vs HCON_00114930 (*Haemonchus contortus*)


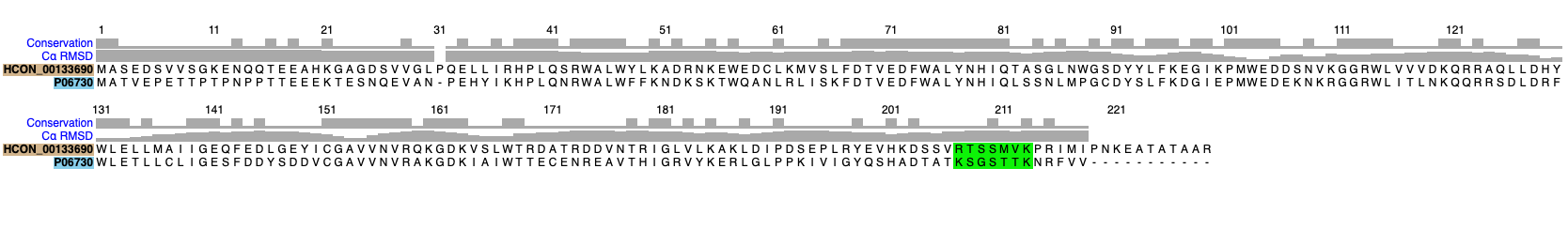

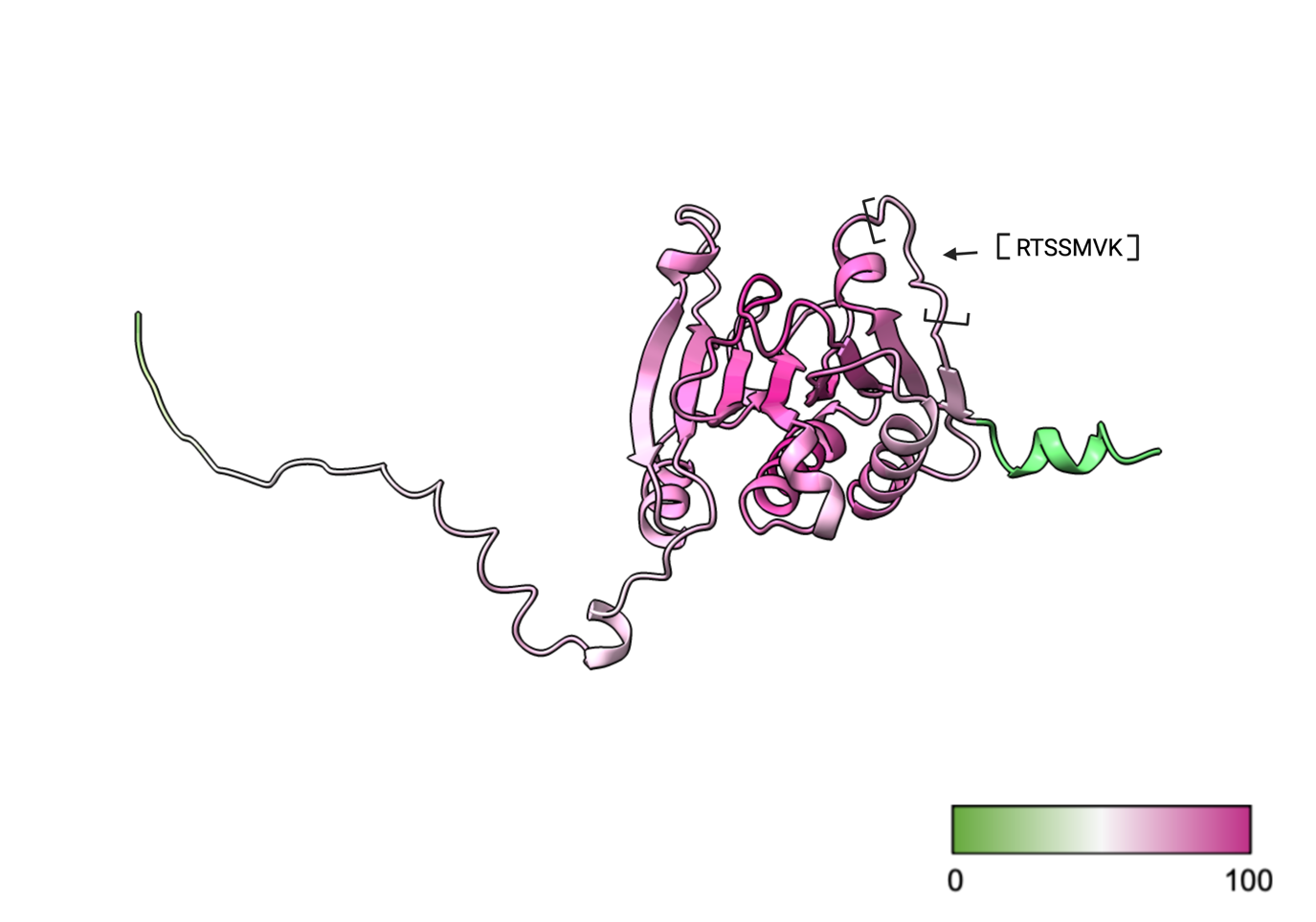
 P06730 (*Homo sapiens*) vs HCON_00133690 (*Haemonchus contortus*)


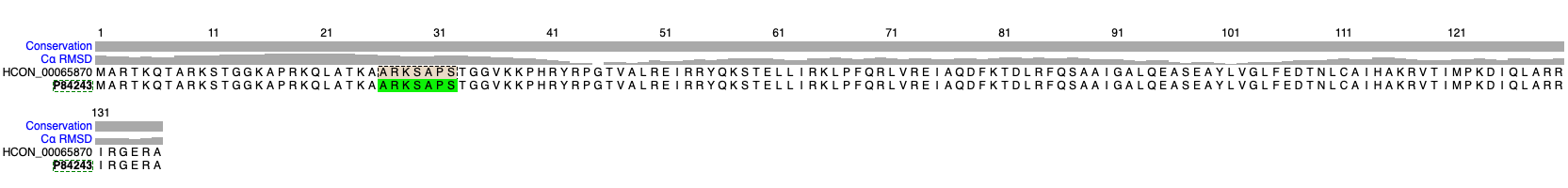

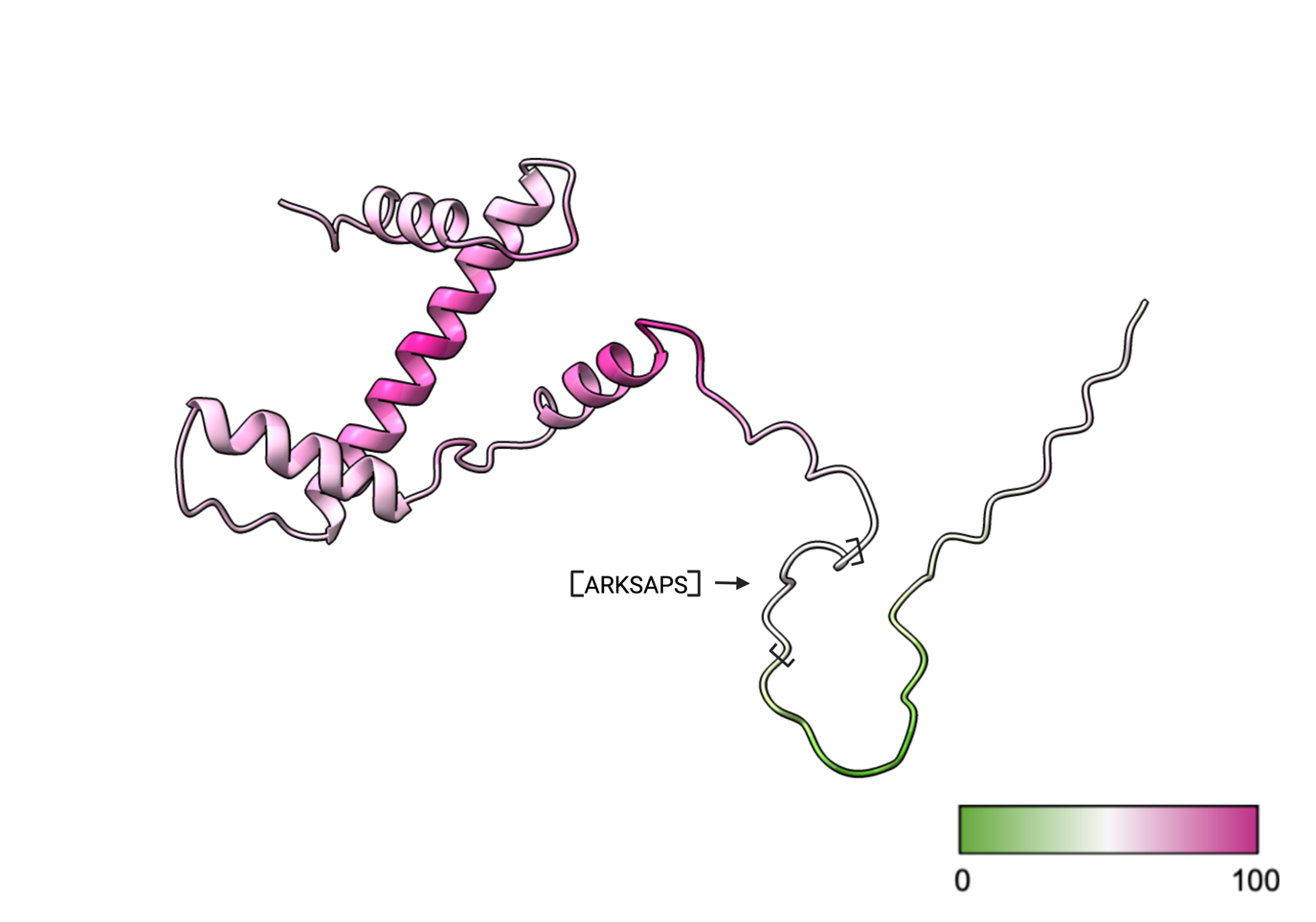
 P84243 (*Homo sapiens*) vs HCON_00065870 (*Haemonchus contortus*)


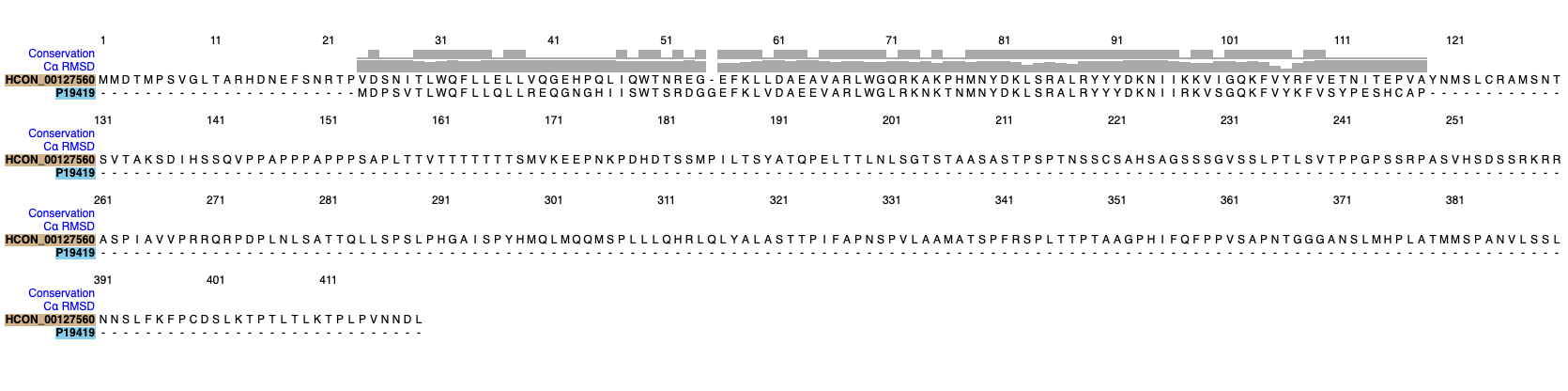

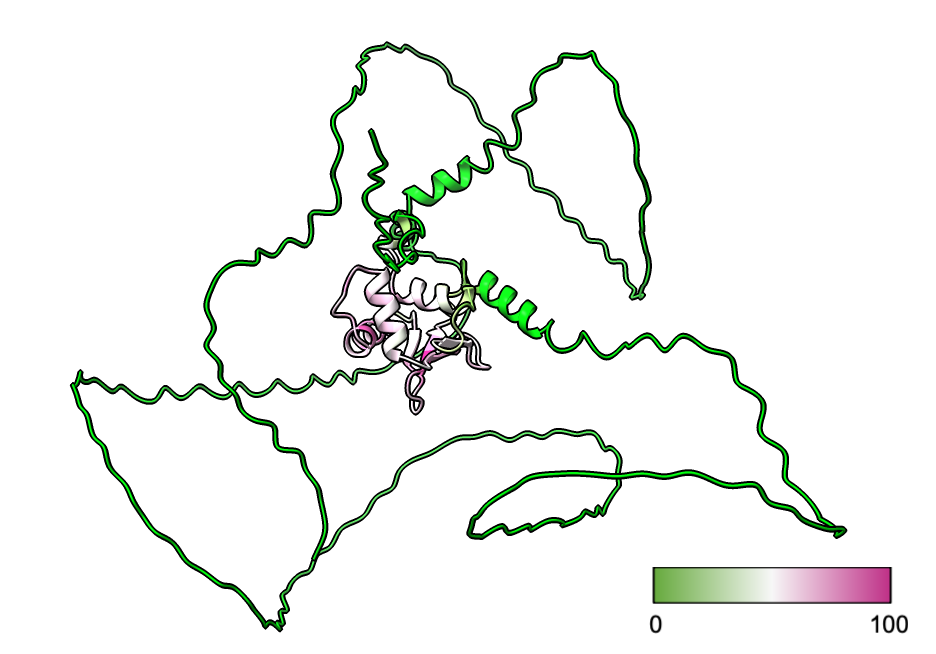
 P19419 (*Homo sapiens*) vs HCON_00127560 (*Haemonchus contortus*)


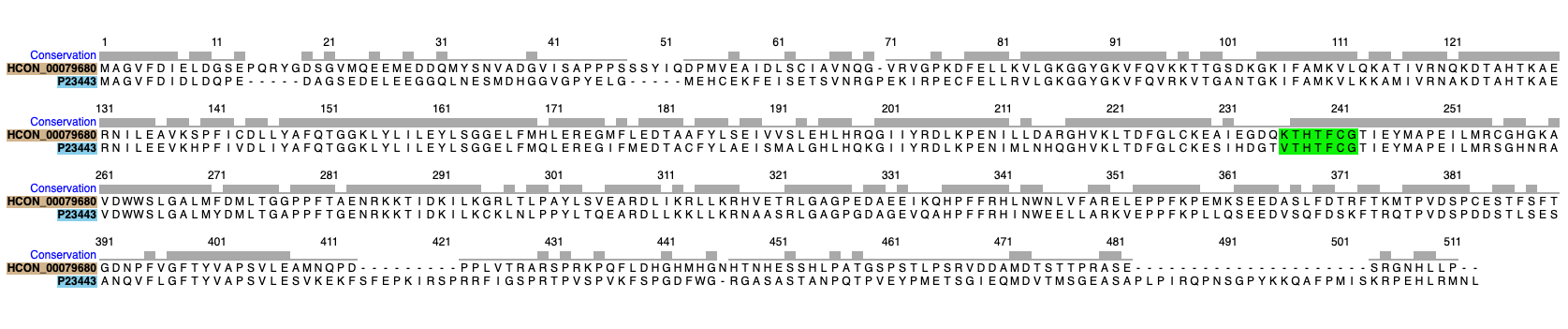

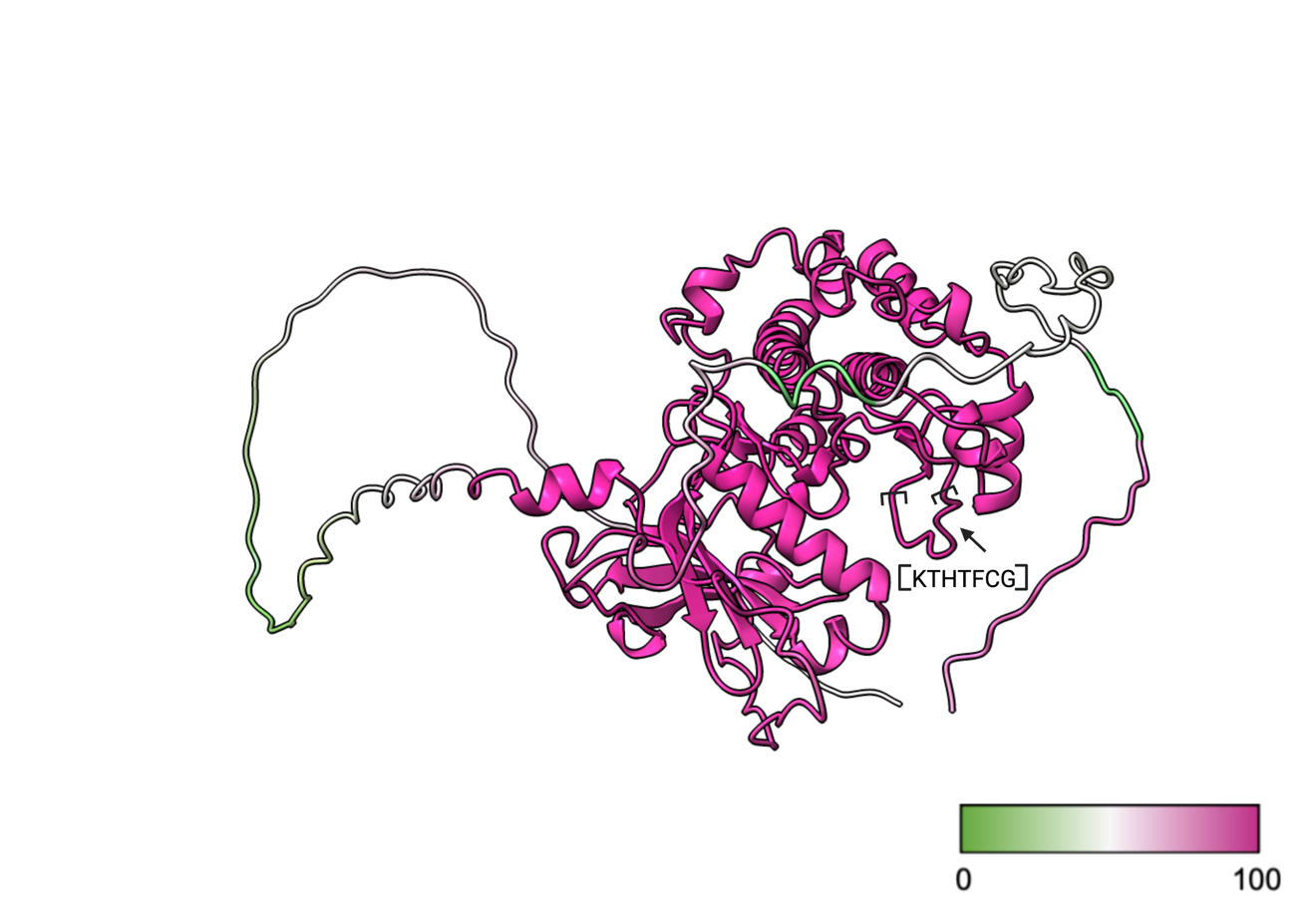
 P23443 (*Homo sapiens*) vs HCON_00079680 (*Haemonchus contortus*)


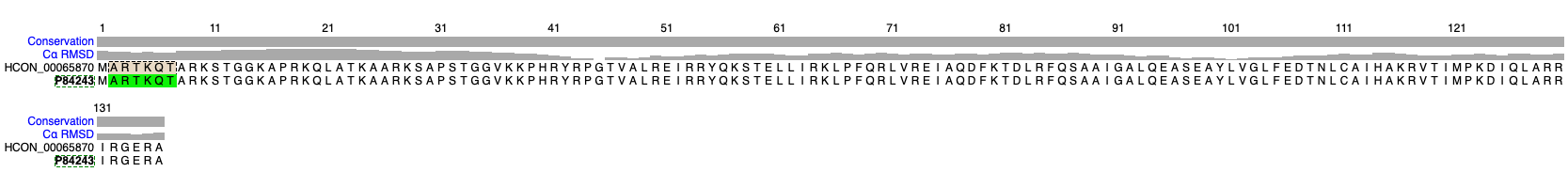

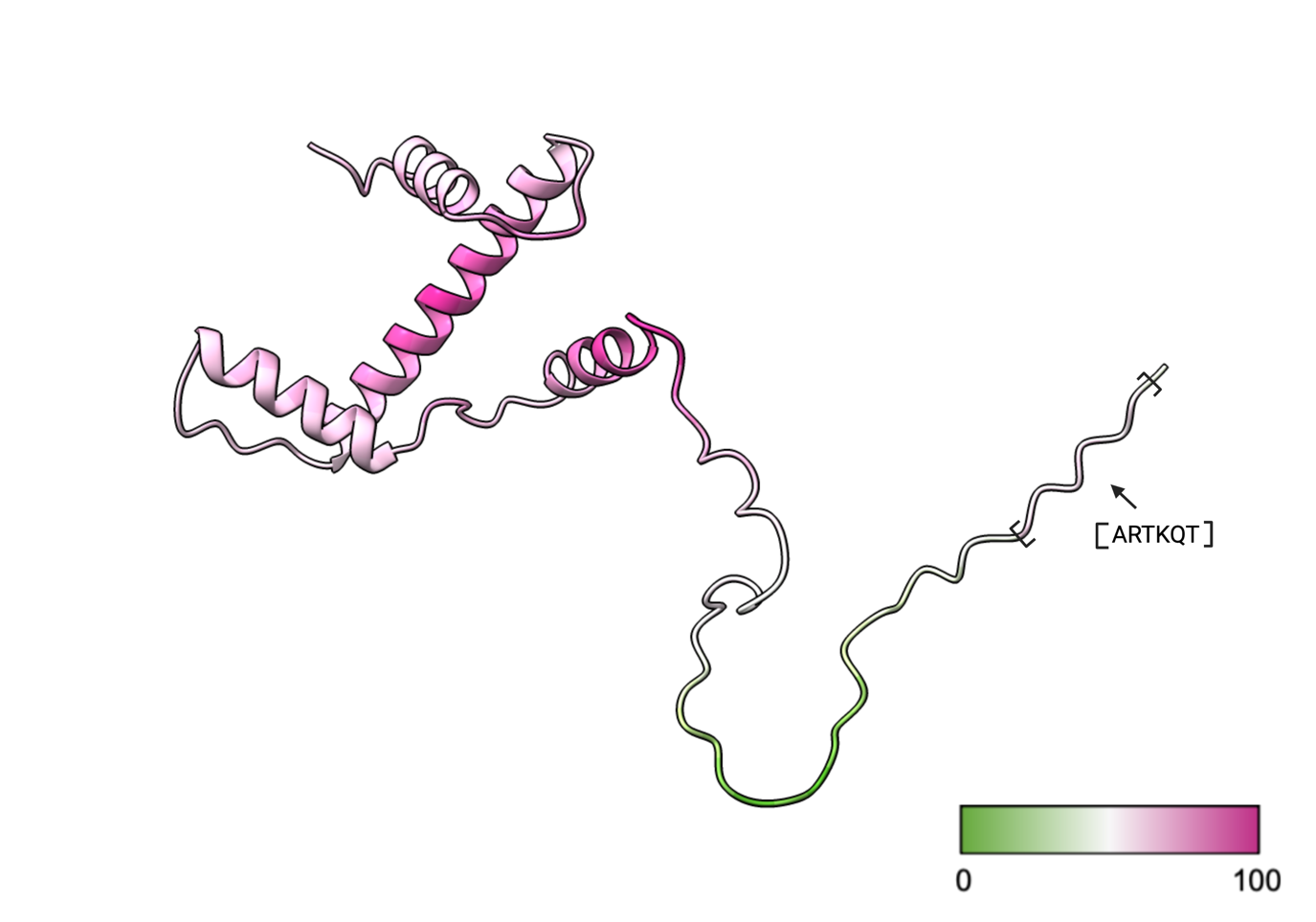
 P84243 (*Homo sapiens*) vs HCON_00065870 (*Haemonchus contortus*)


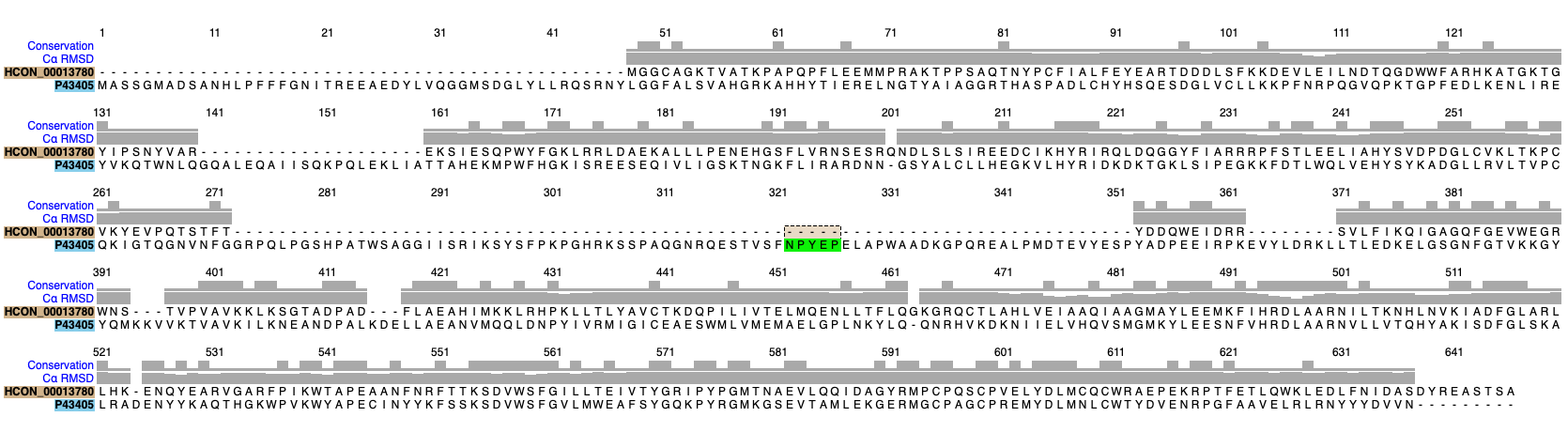

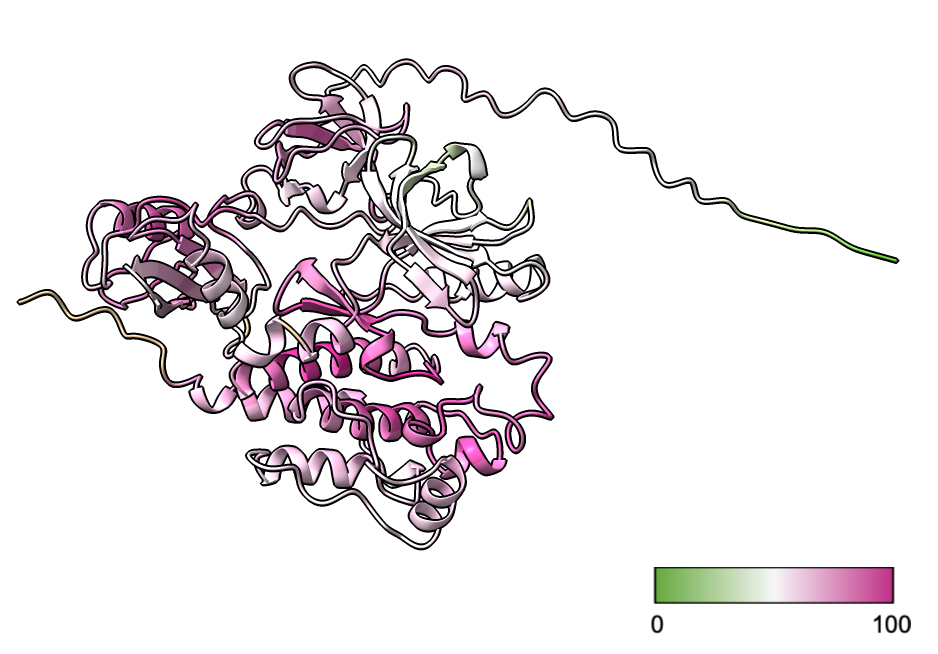
 P43405 (*Homo sapiens*) vs HCON_00013780 (*Haemonchus contortus*)


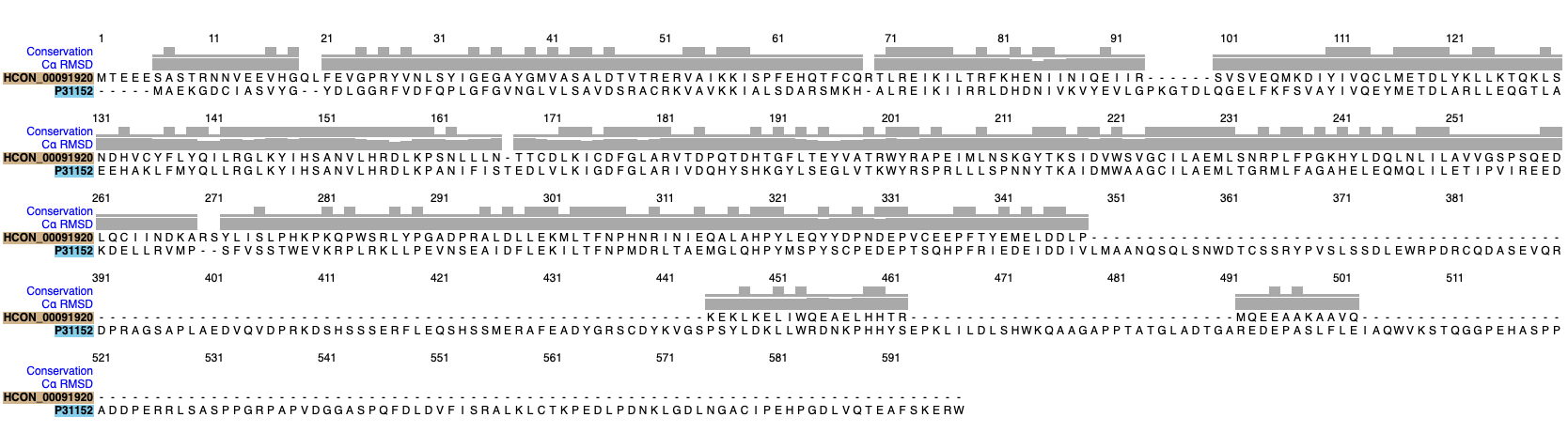

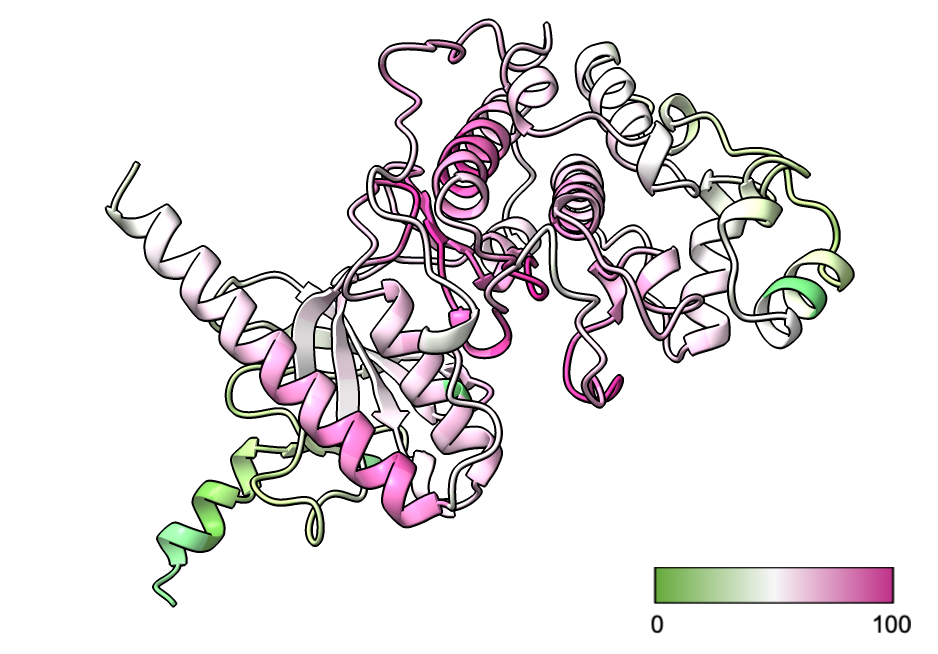
 P31152 (*Homo sapiens*) vs HCON_00091920 (*Haemonchus contortus*)


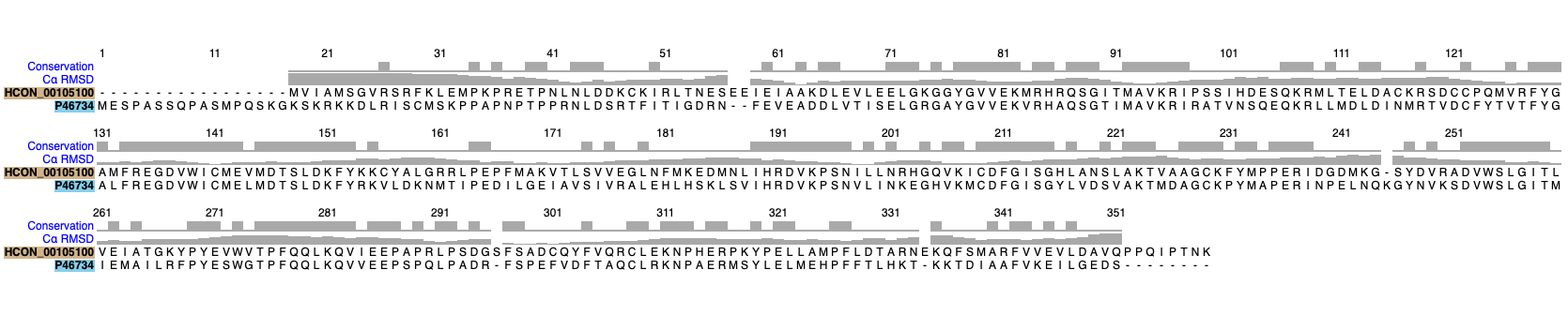

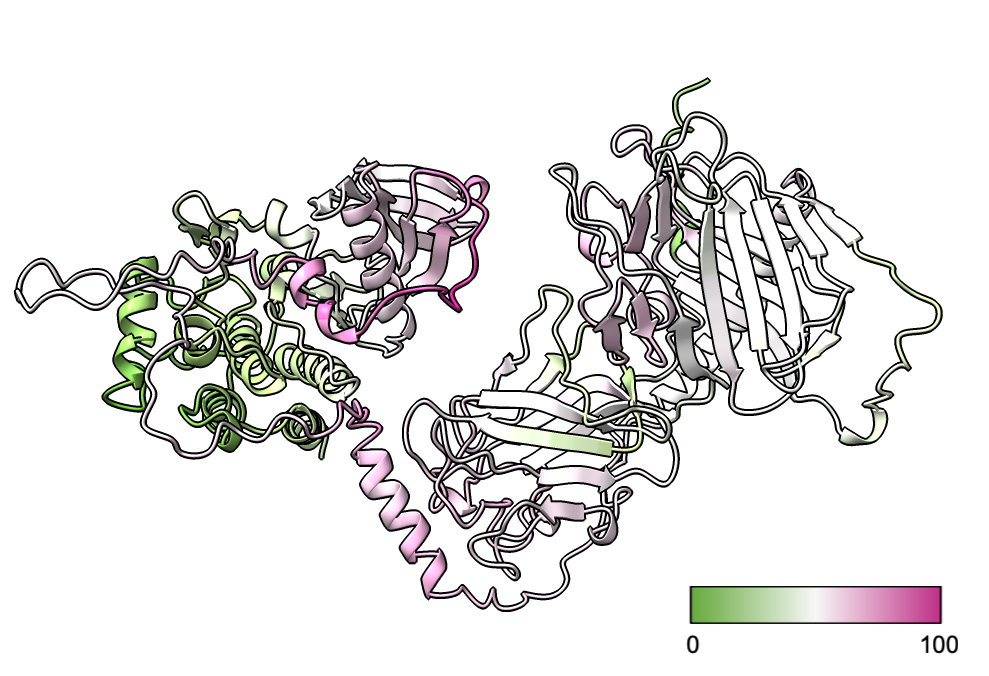
 O60674 (*Homo sapiens*) vs HCON_00109060 (*Haemonchus contortus*)


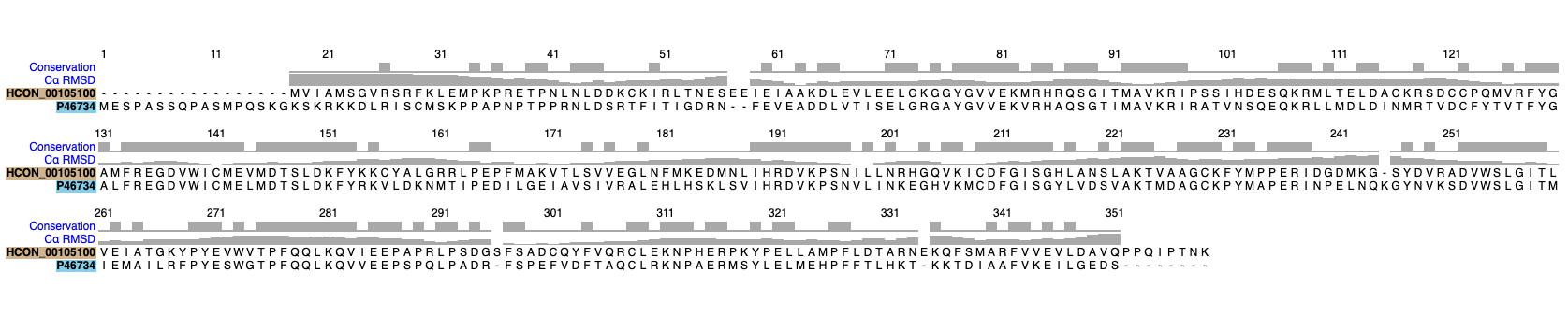

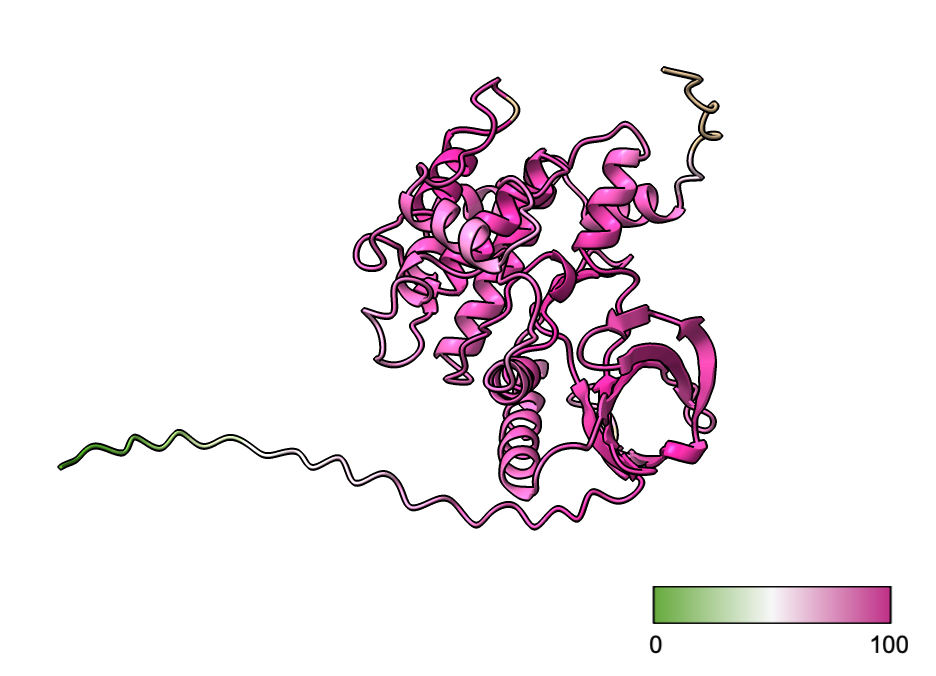
 P46734 (*Homo sapiens*) vs HCON_00105100 (*Haemonchus contortus*)


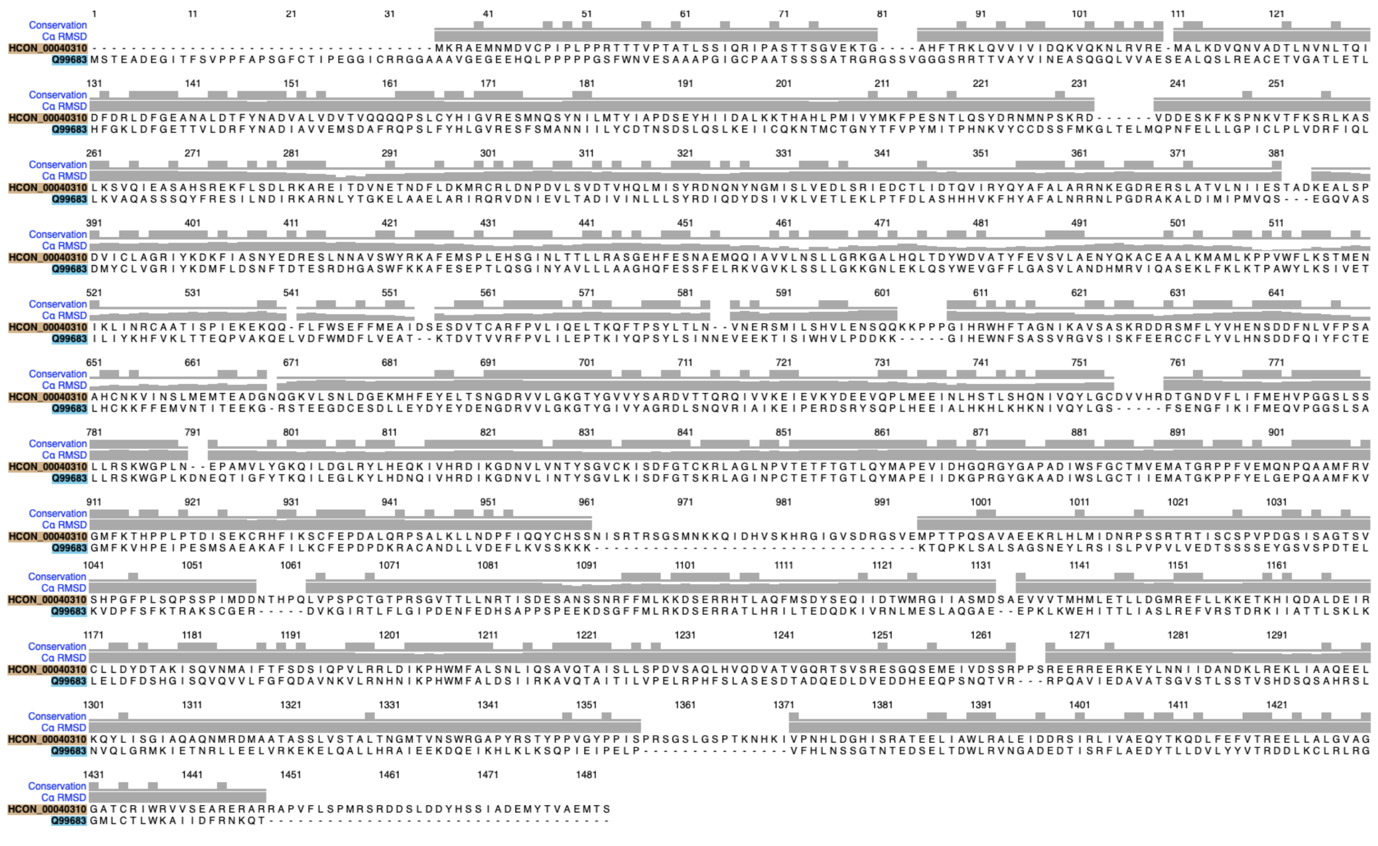

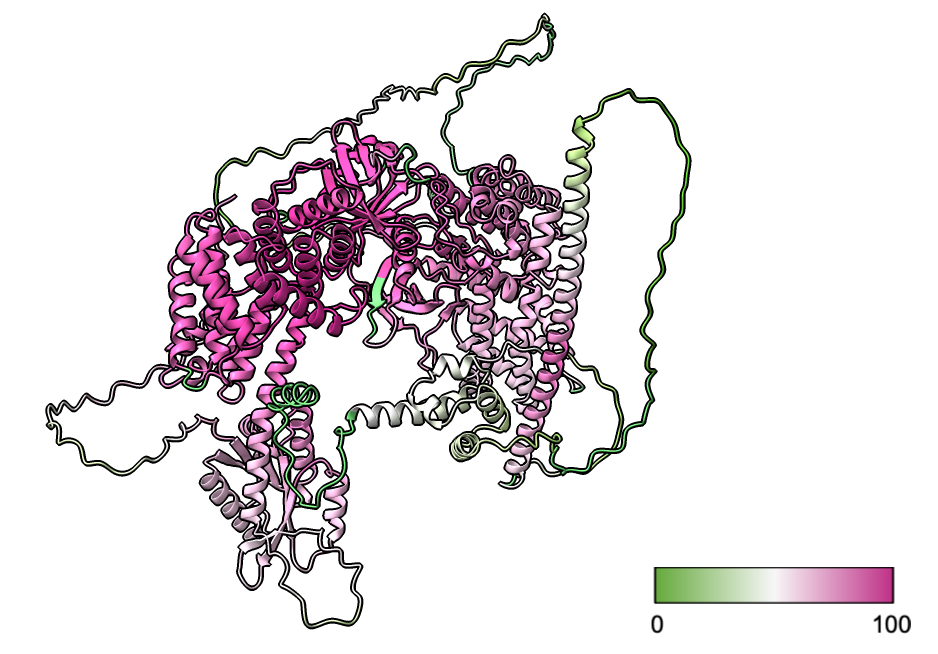
 Q99683 (*Homo sapiens*) vs HCON_00040310 (*Haemonchus contortus*)


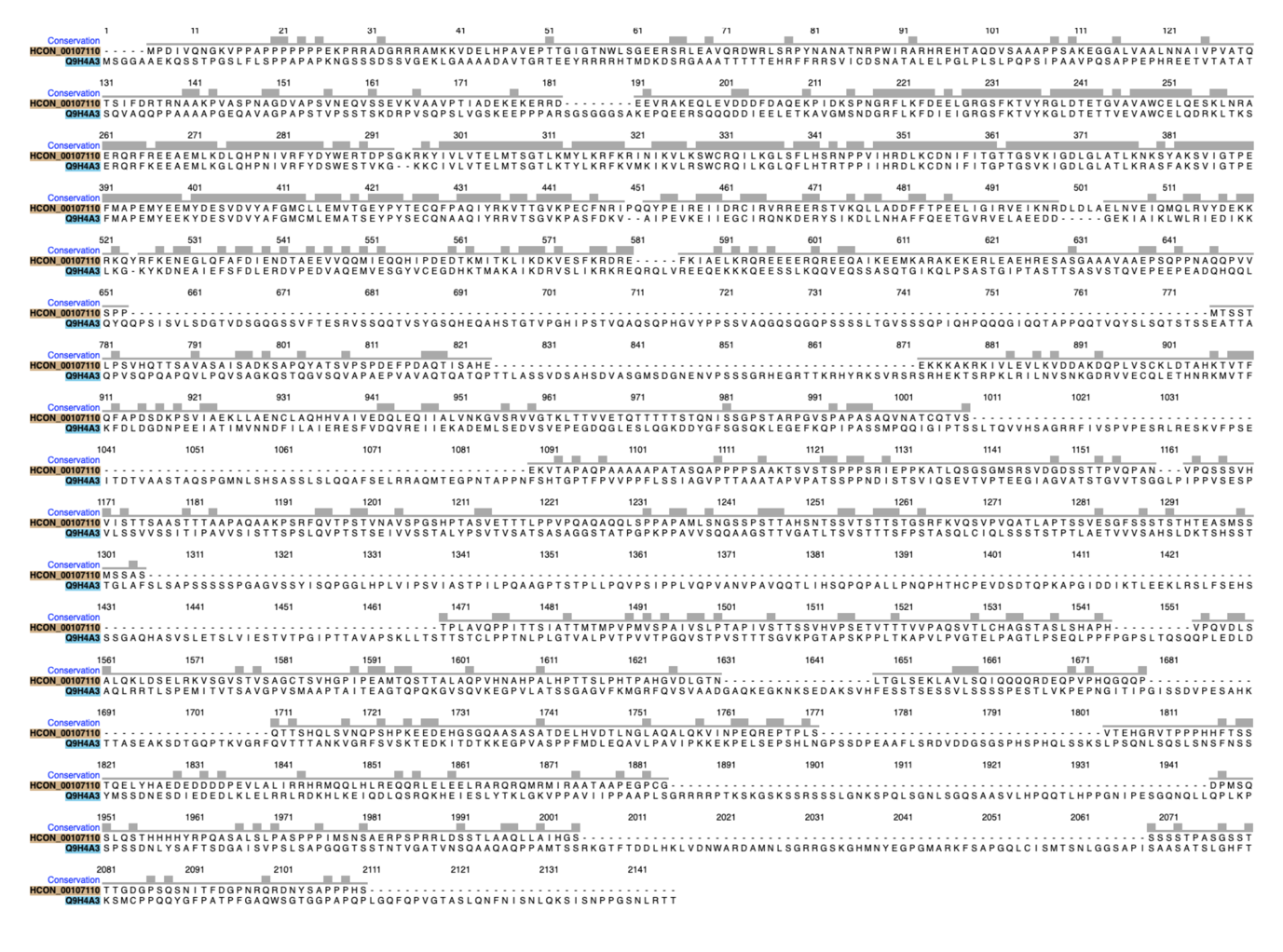


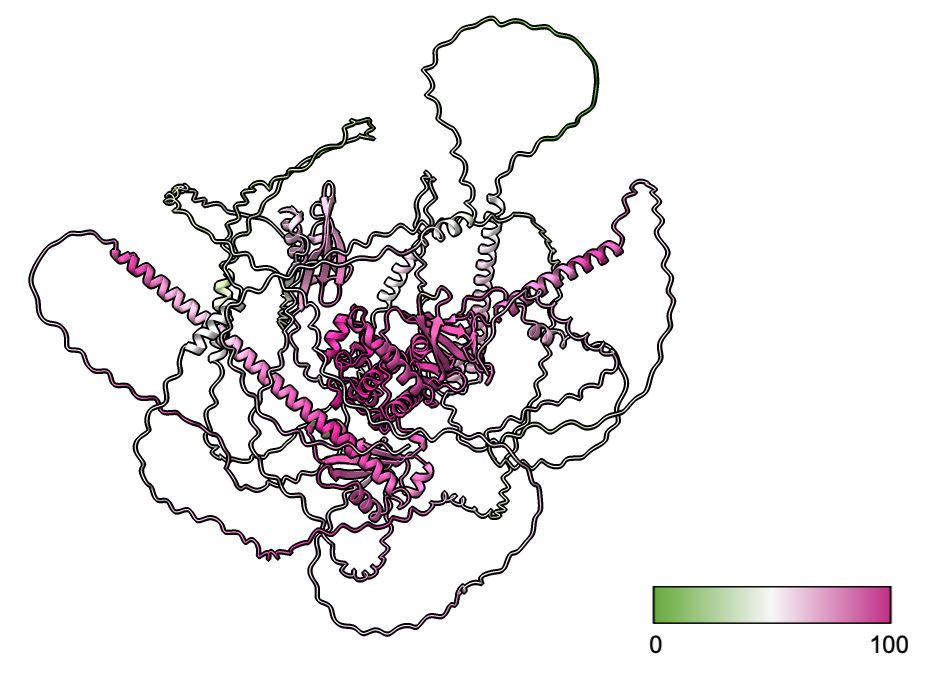
 Q9H4A3 (*Homo sapiens*) vs HCON_00107110 (*Haemonchus contortus*)


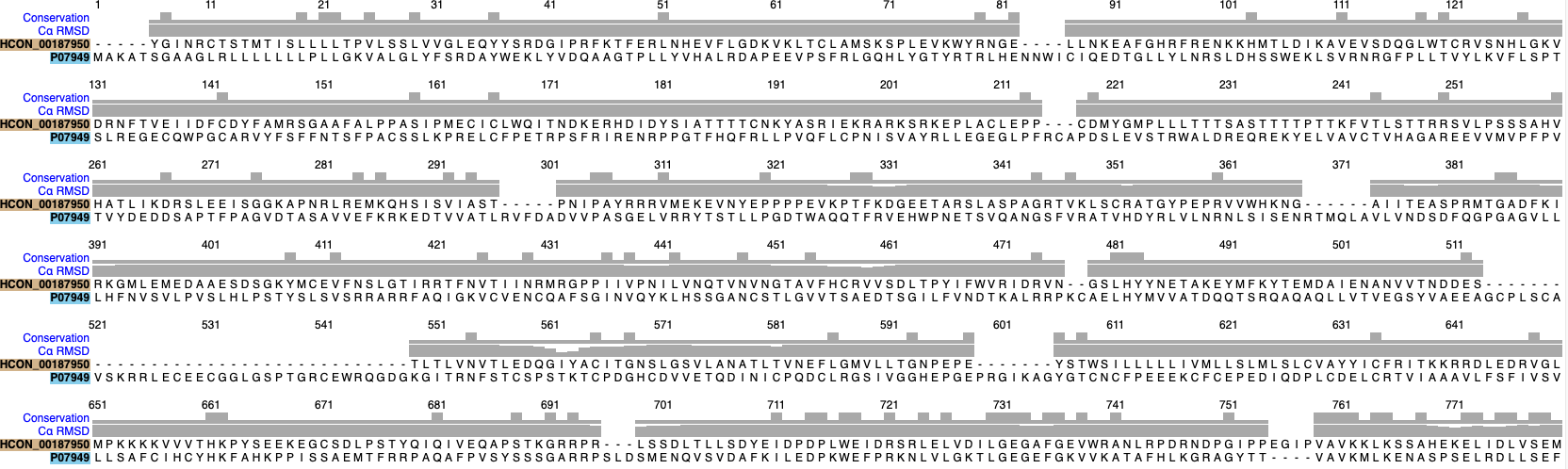


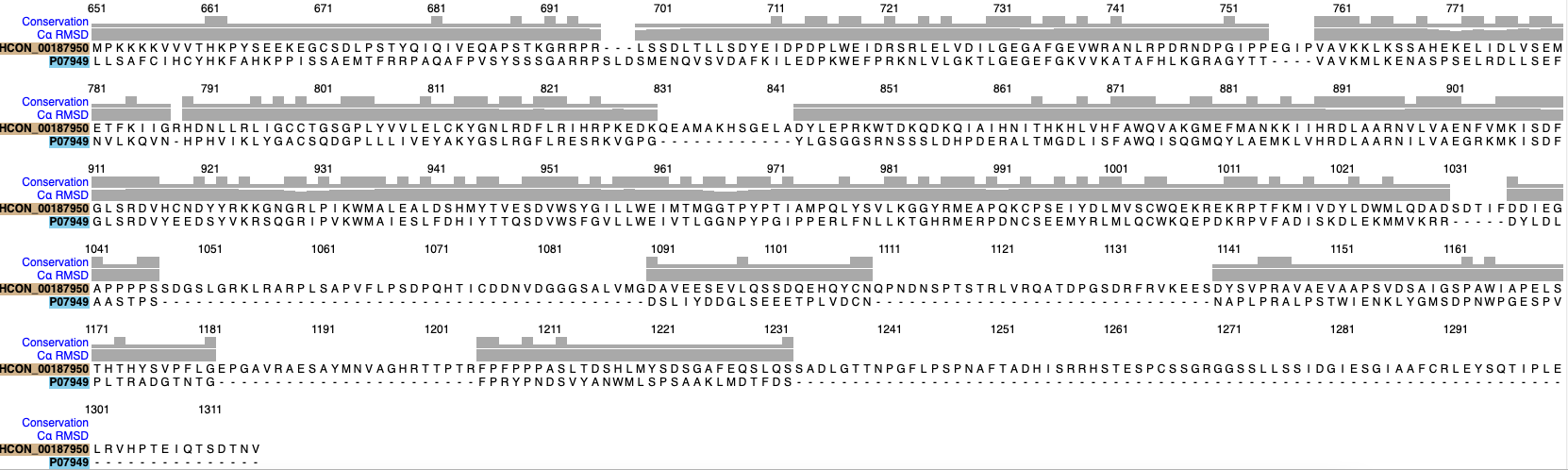


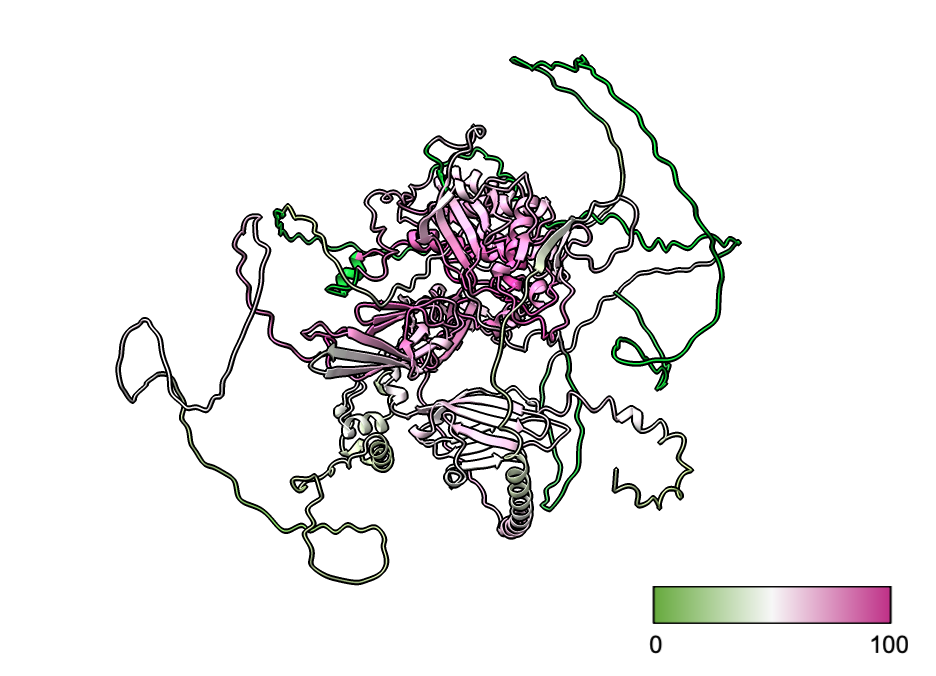
 P07949 (*Homo sapiens*) vs HCON_00187950 (*Haemonchus contortus*)


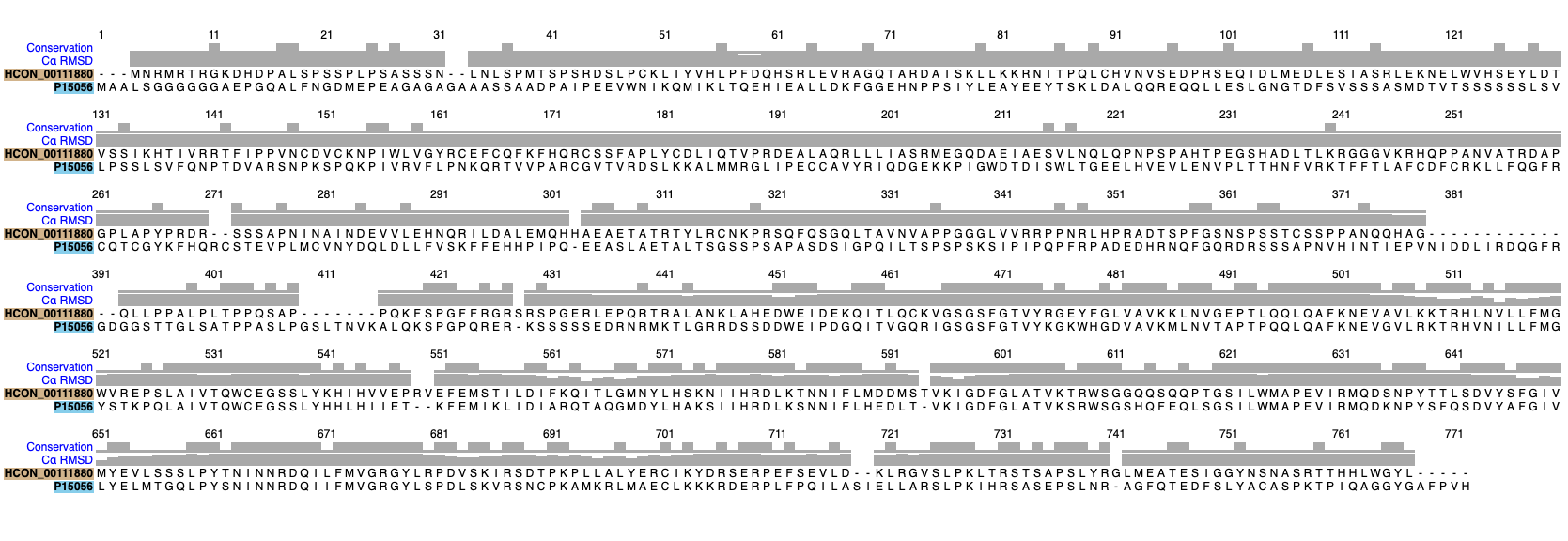


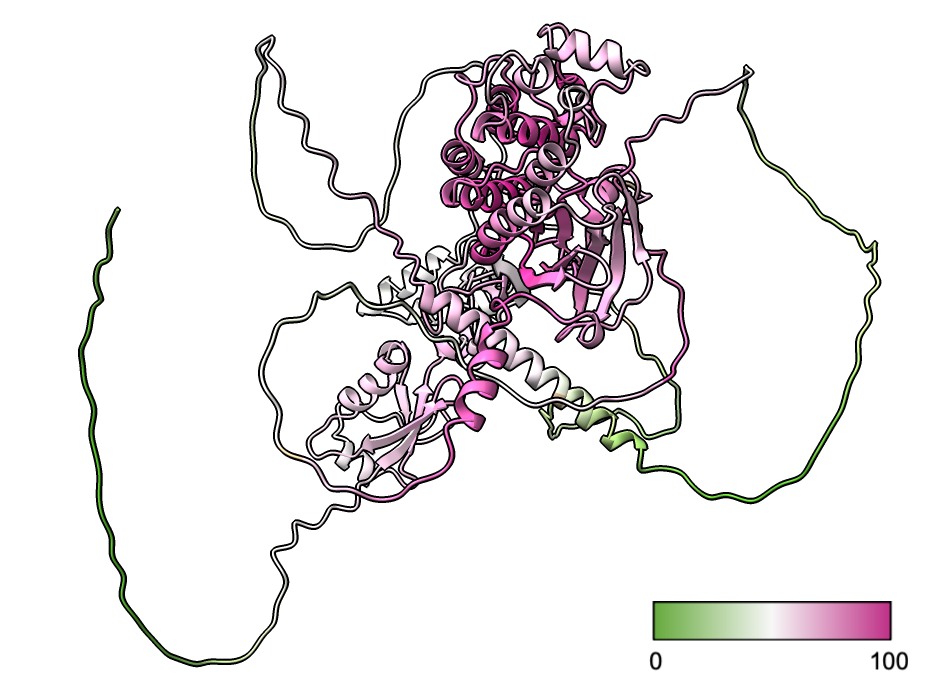
 P15056 (*Homo sapiens*) vs HCON_00111880 (*Haemonchus contortus*)


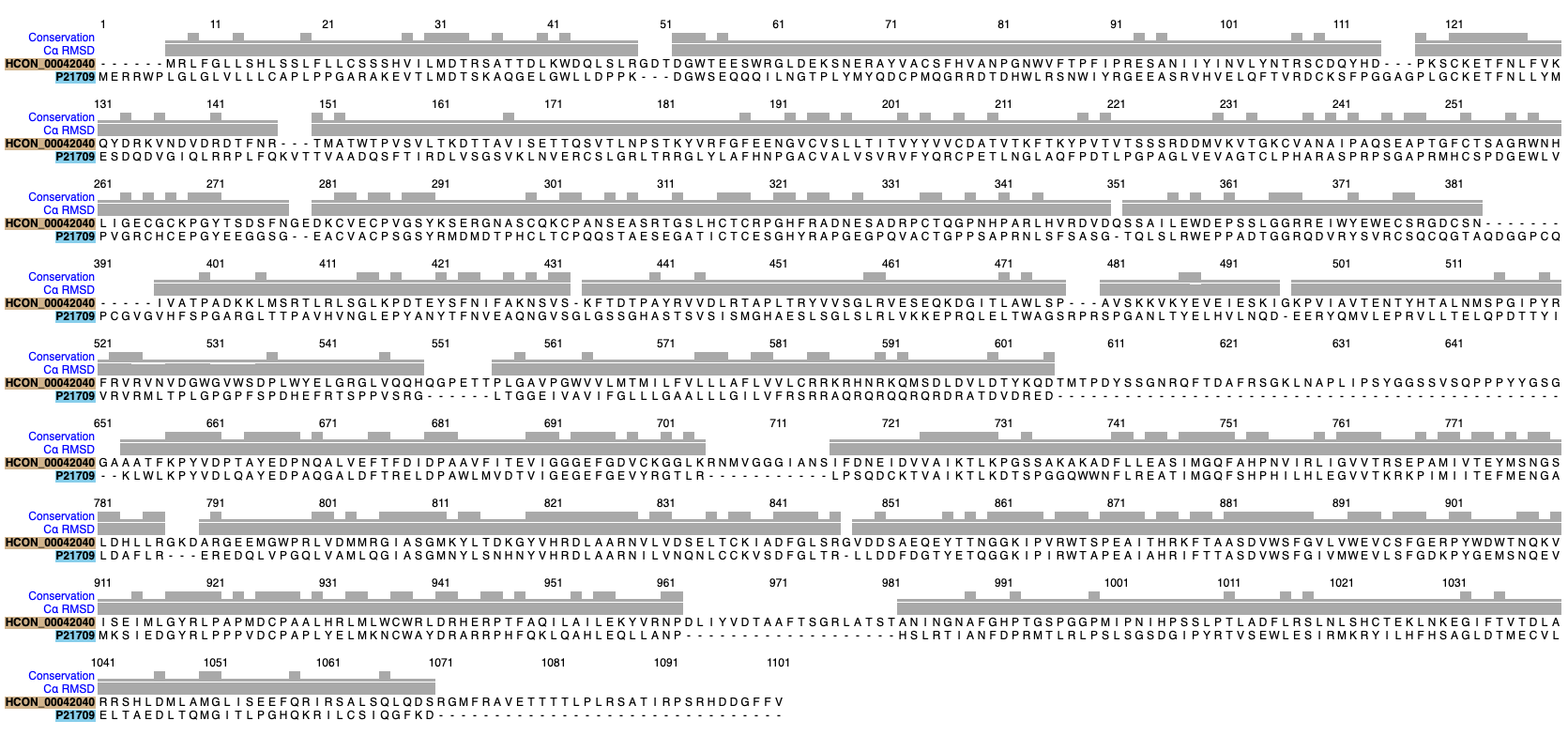

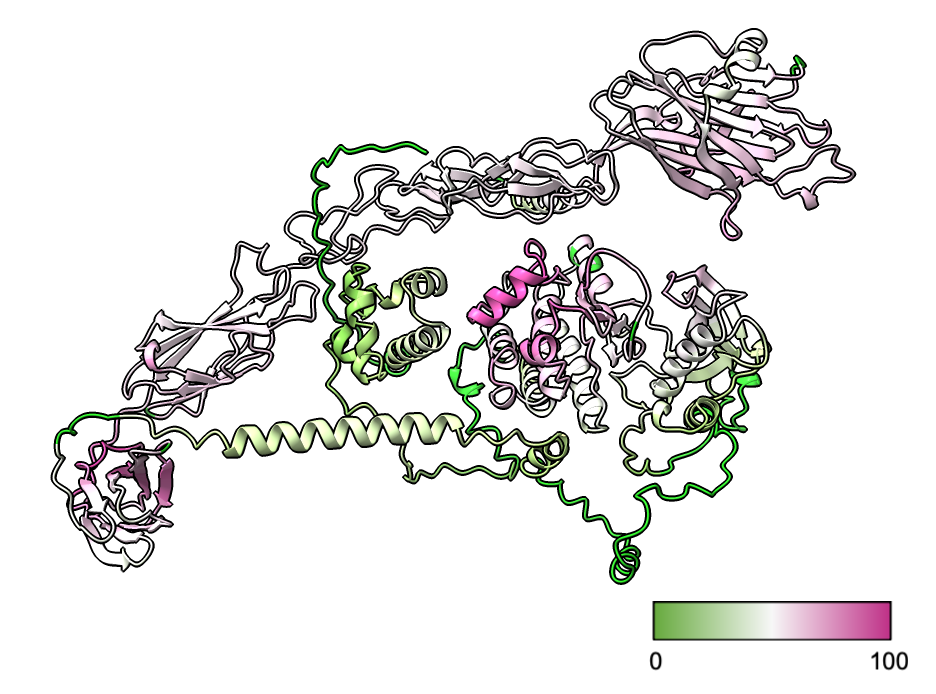
 P21709 (*Homo sapiens*) vs HCON_00042040 (*Haemonchus contortus*)


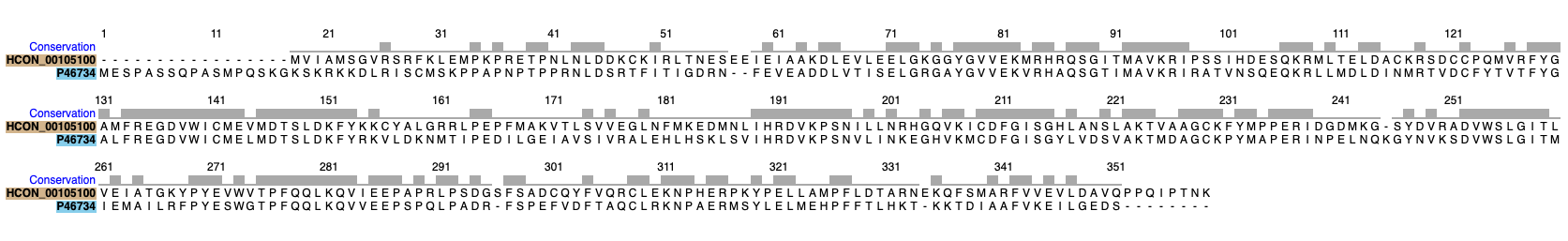


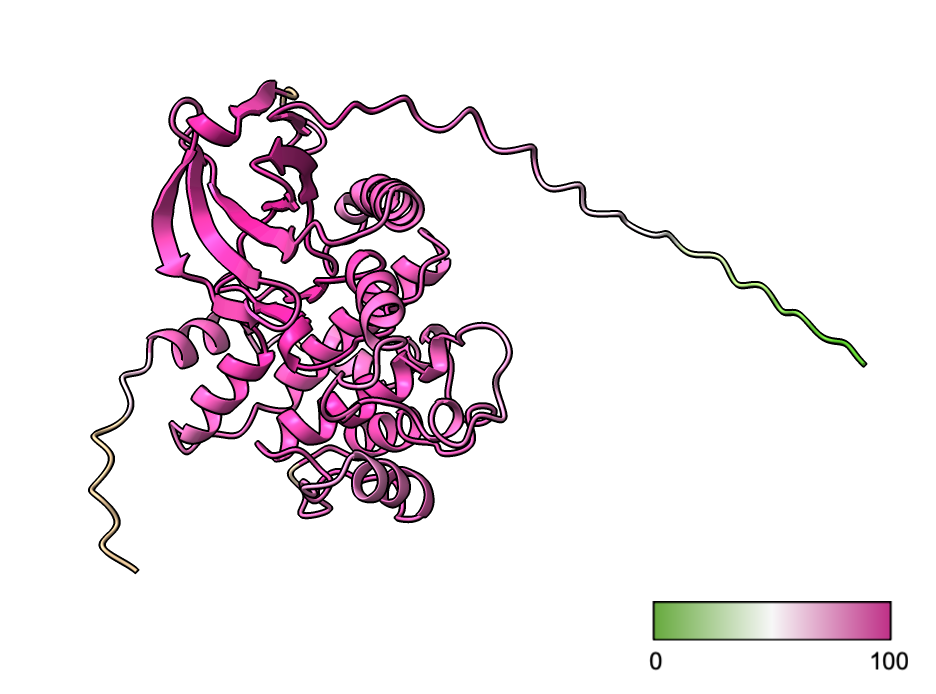
 P46734 (*Homo sapiens*) vs HCON_00105100 (*Haemonchus contortus*)


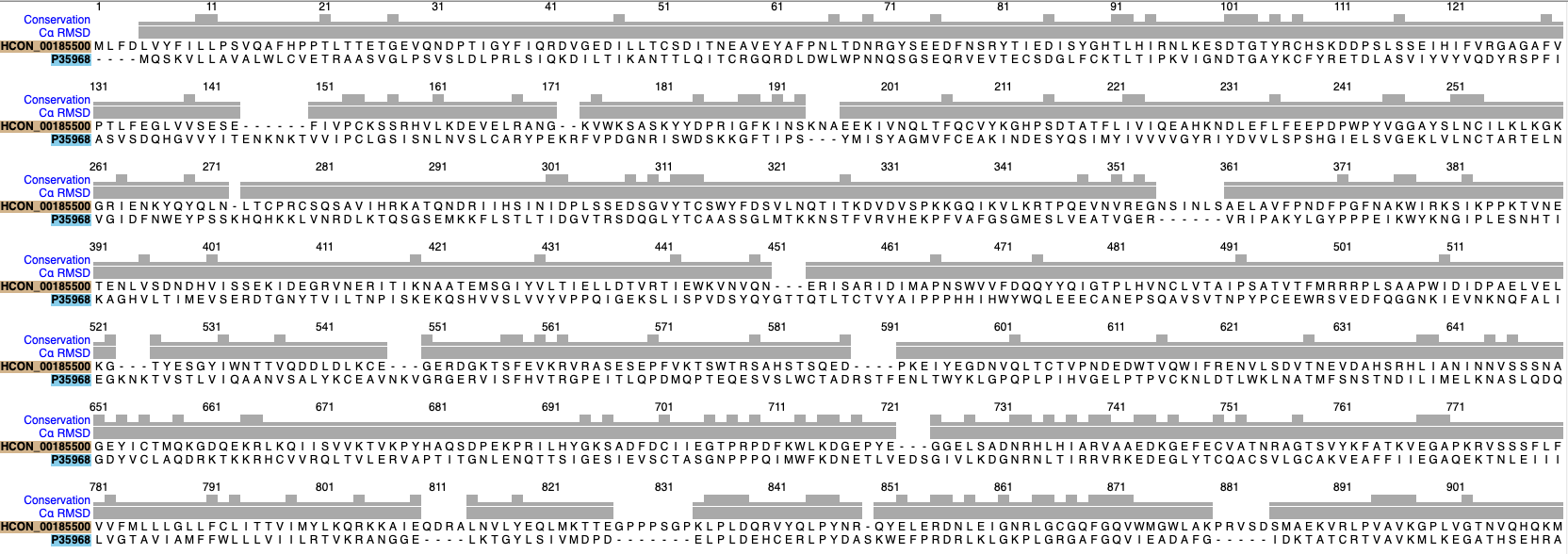


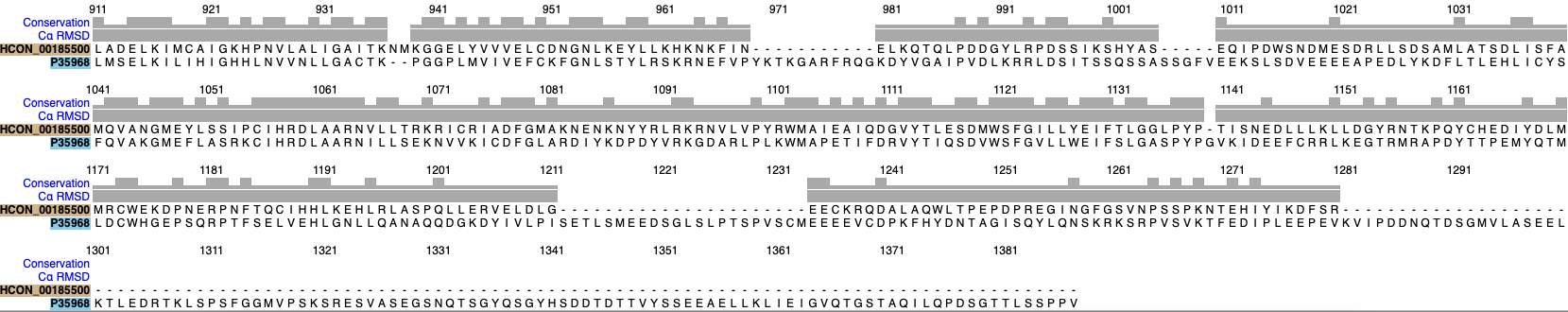


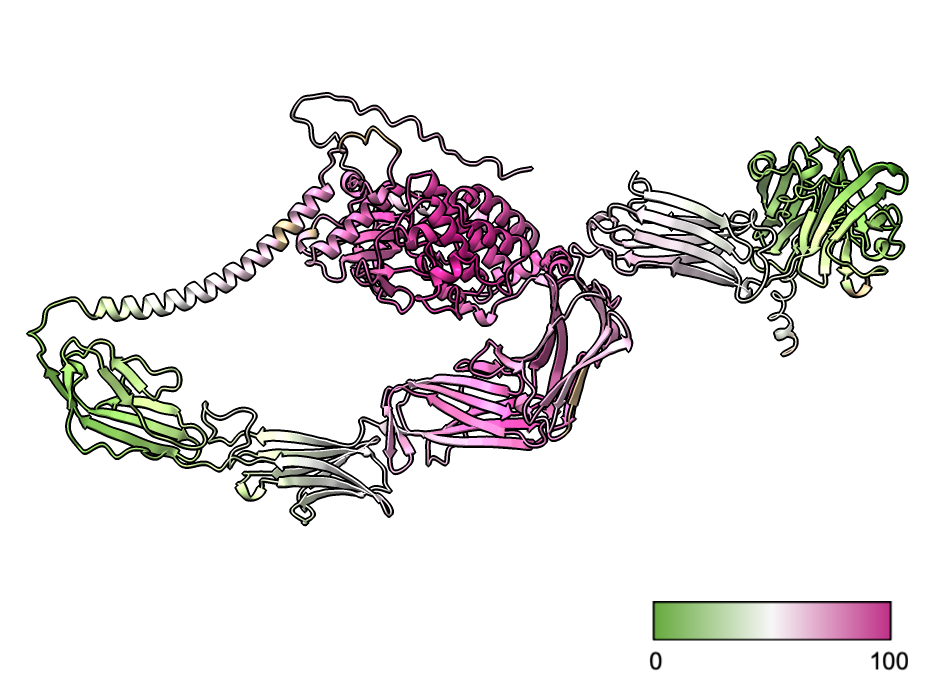
 P35968 (*Homo sapiens*) vs HCON_00185500 (*Haemonchus contortus*)


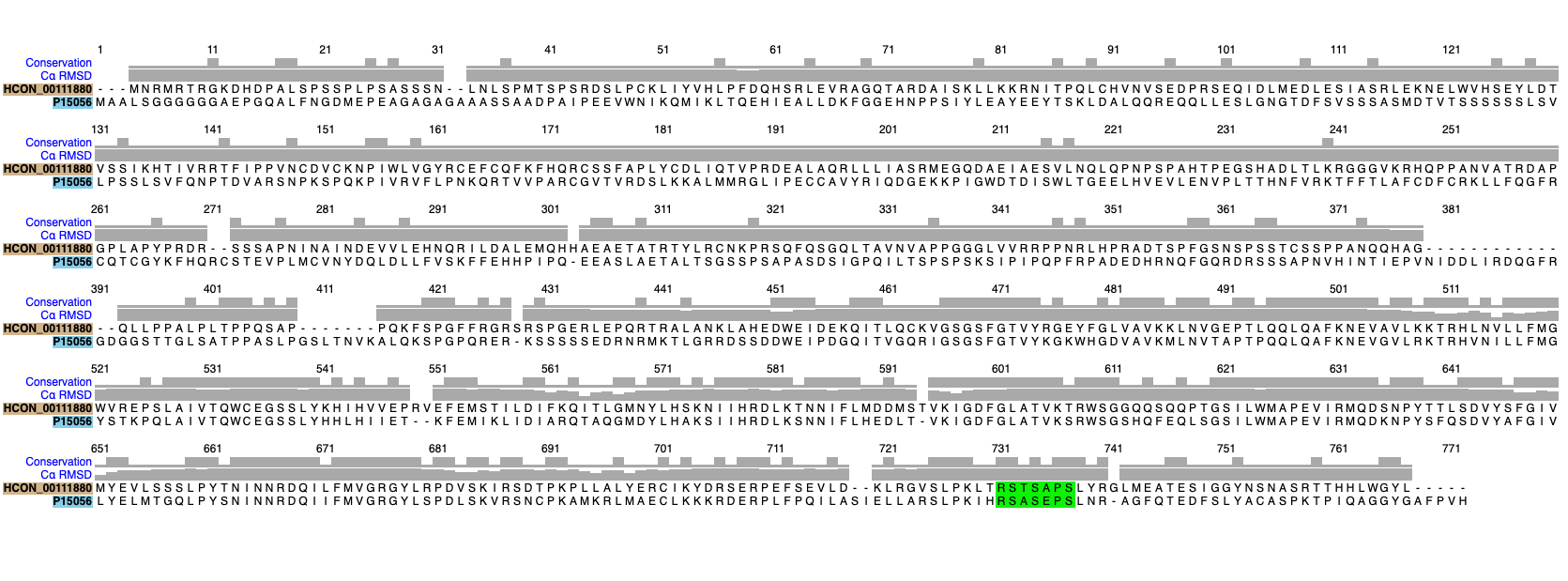


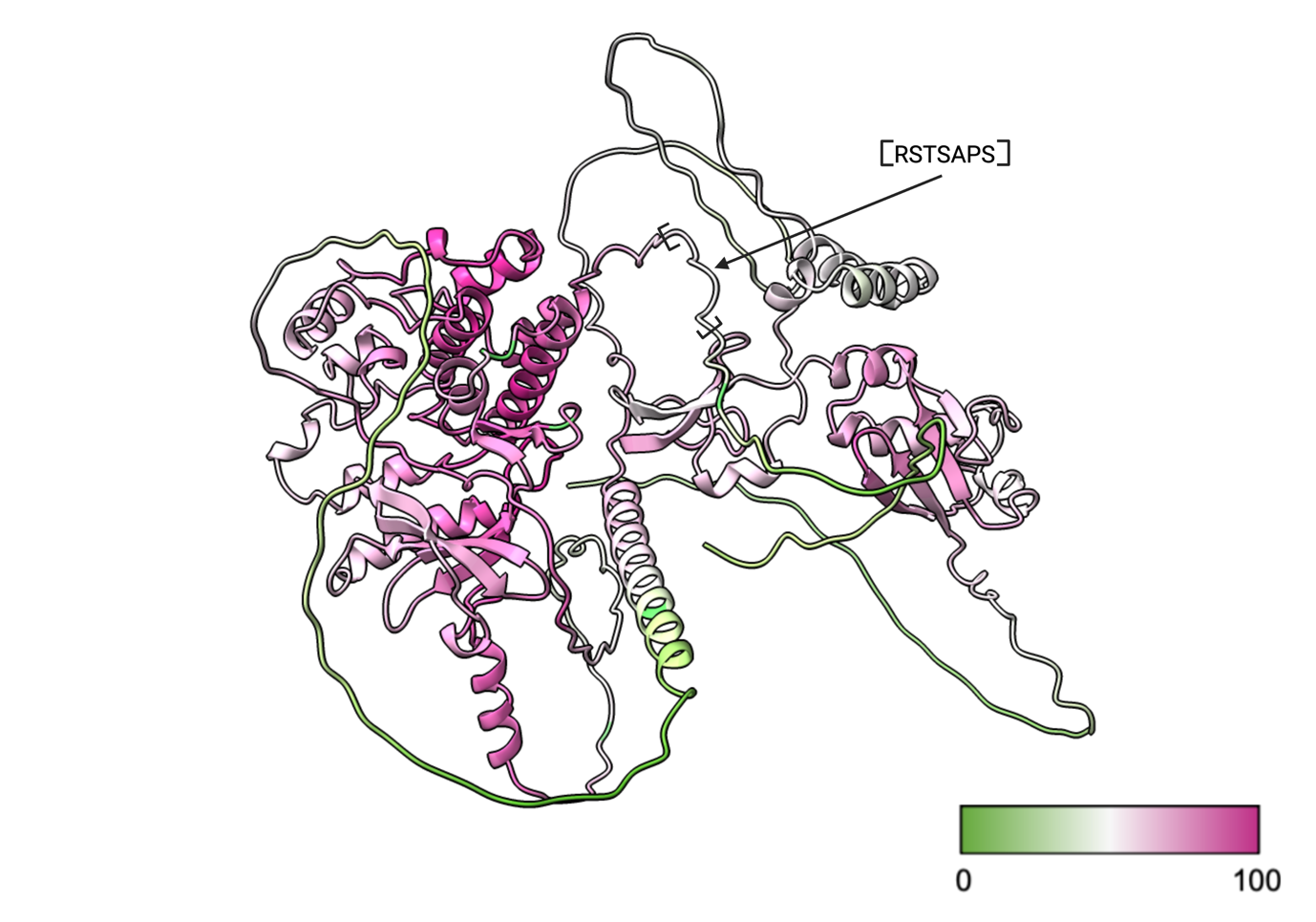
 P15056 (*Homo sapiens*) vs HCON_00111880 (*Haemonchus contortus*)


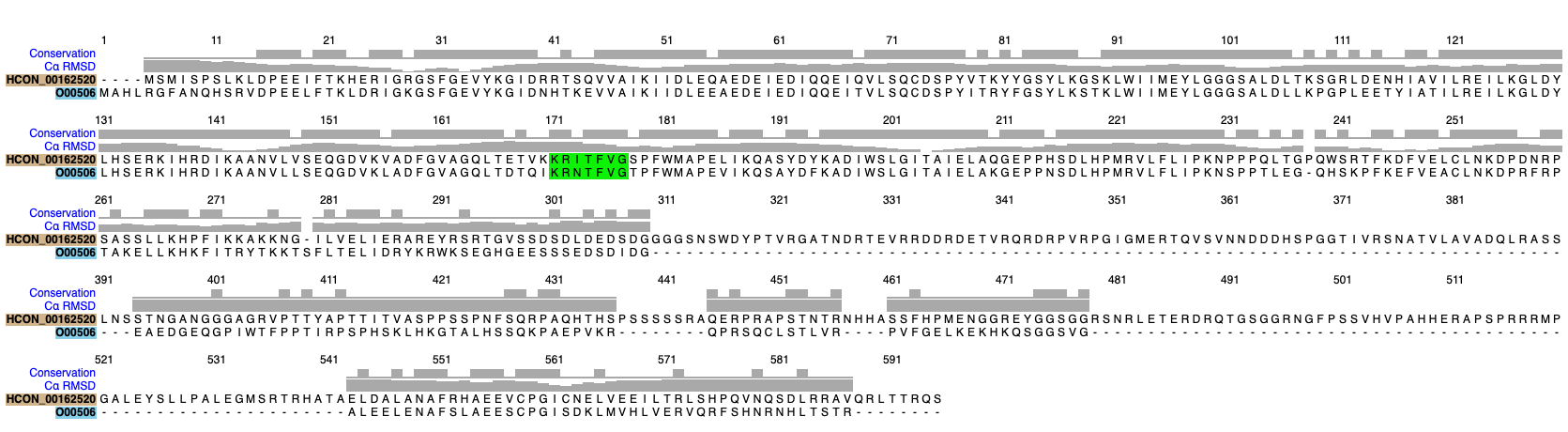


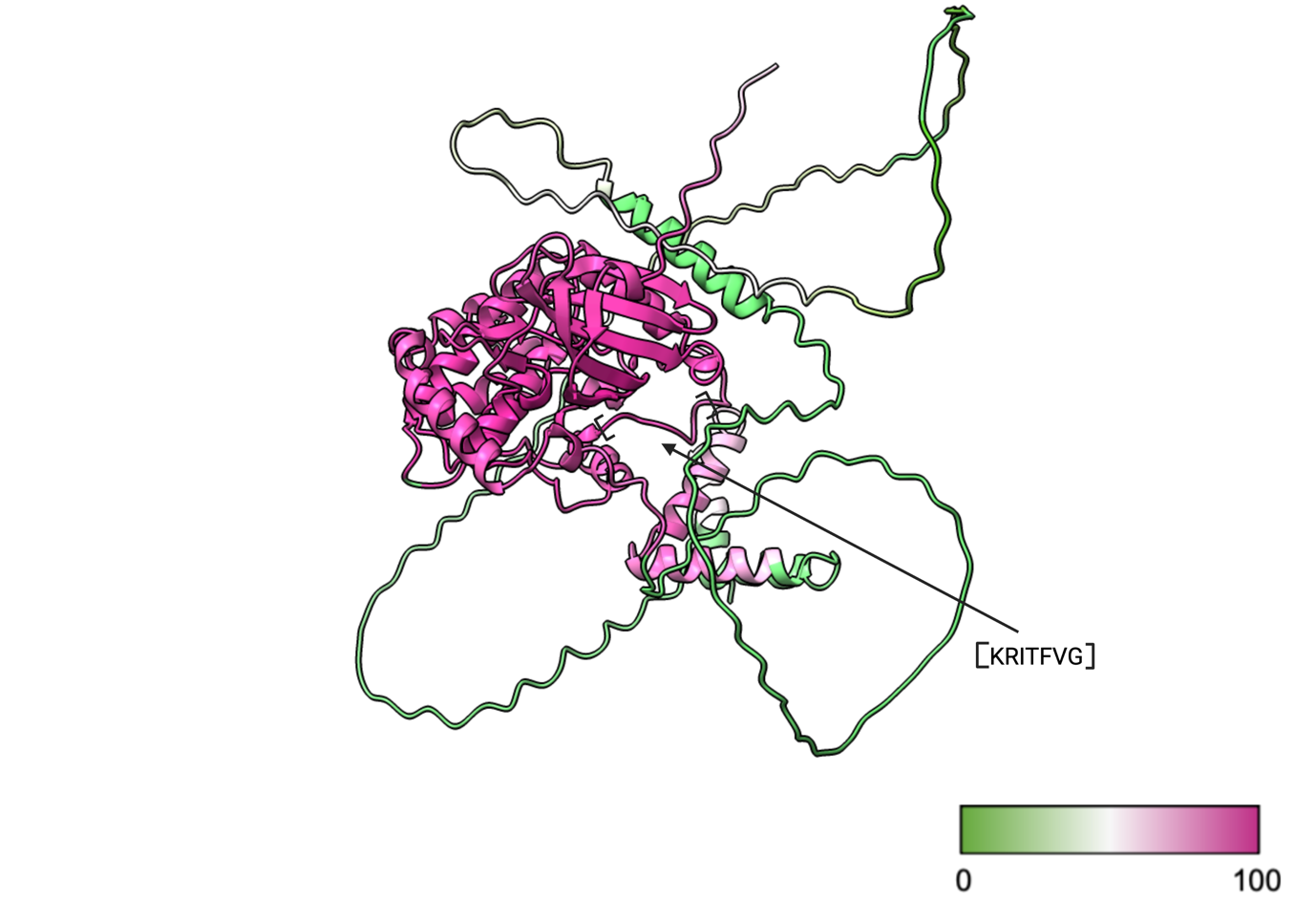


O00506 (*Homo sapiens*) vs HCON_00162520 (*Haemonchus contortus*)


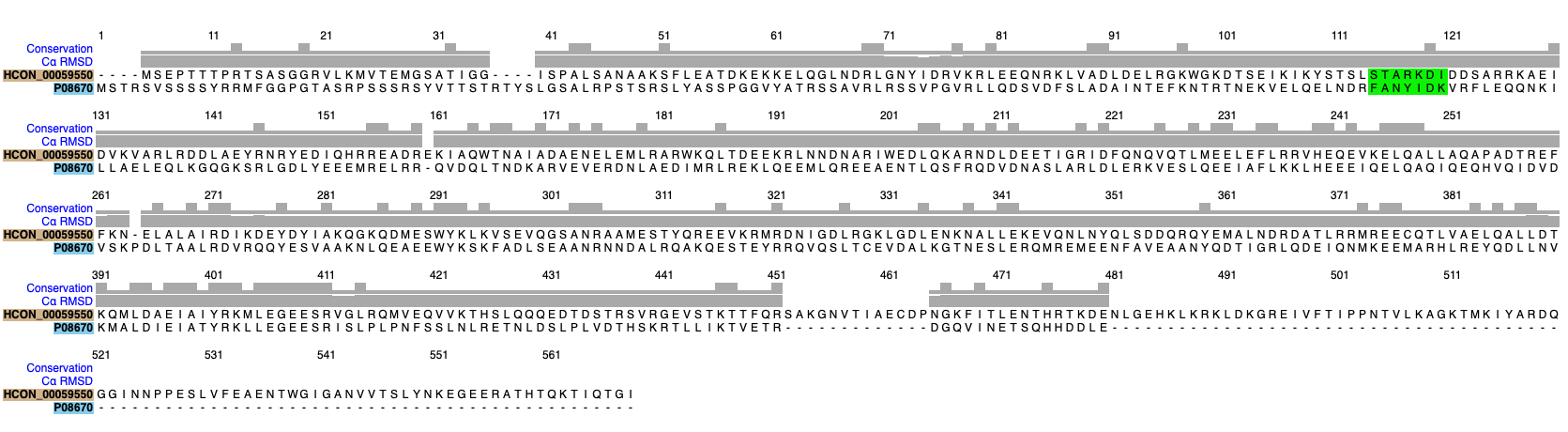


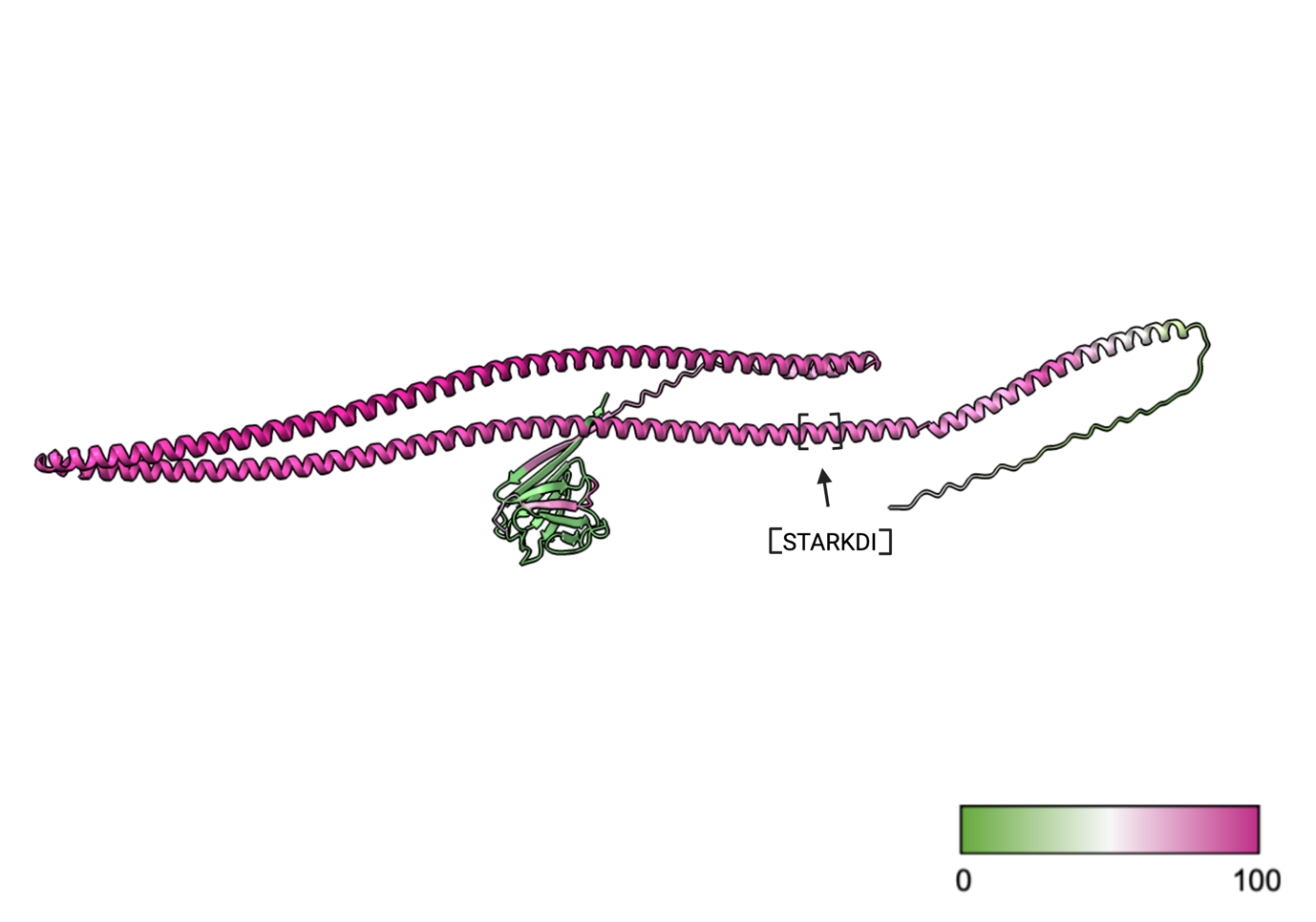
 P08670 (*Homo sapiens*) vs HCON_00059550 (*Haemonchus contortus*)


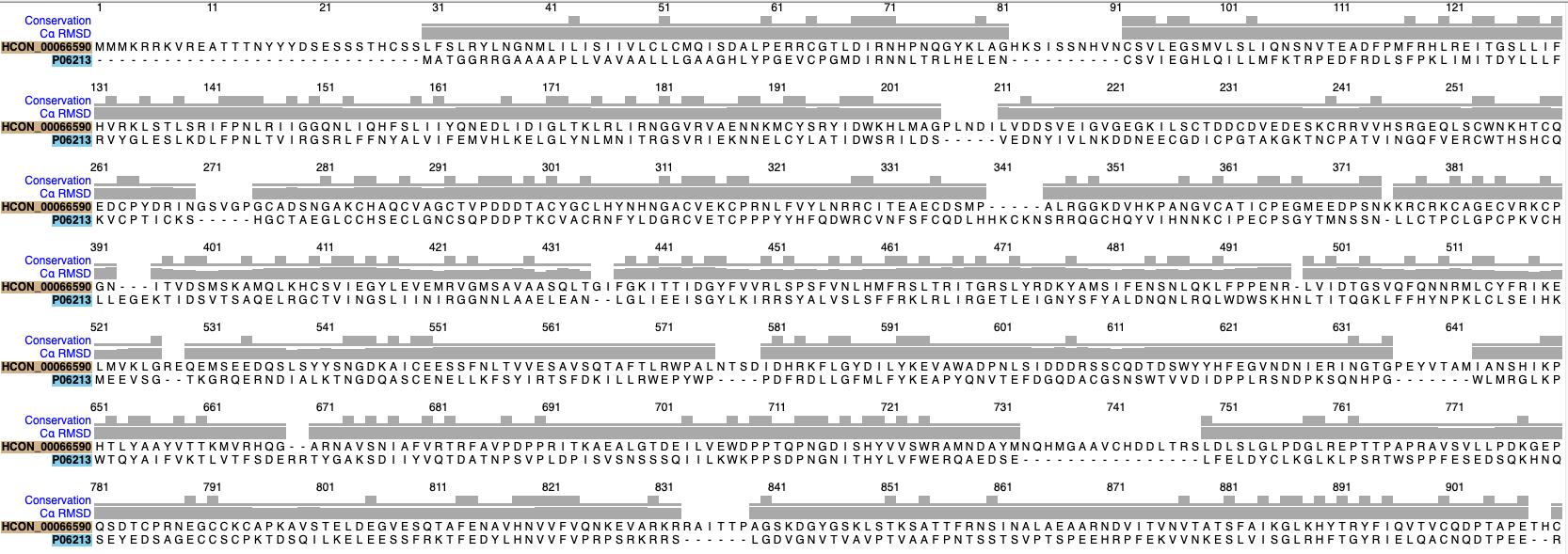


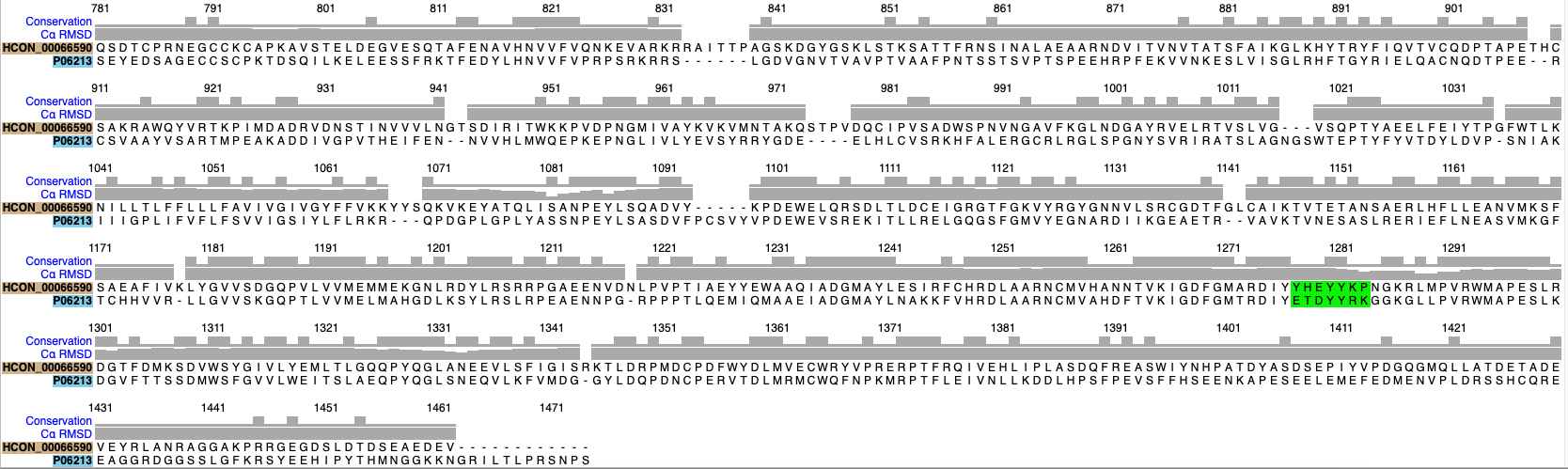


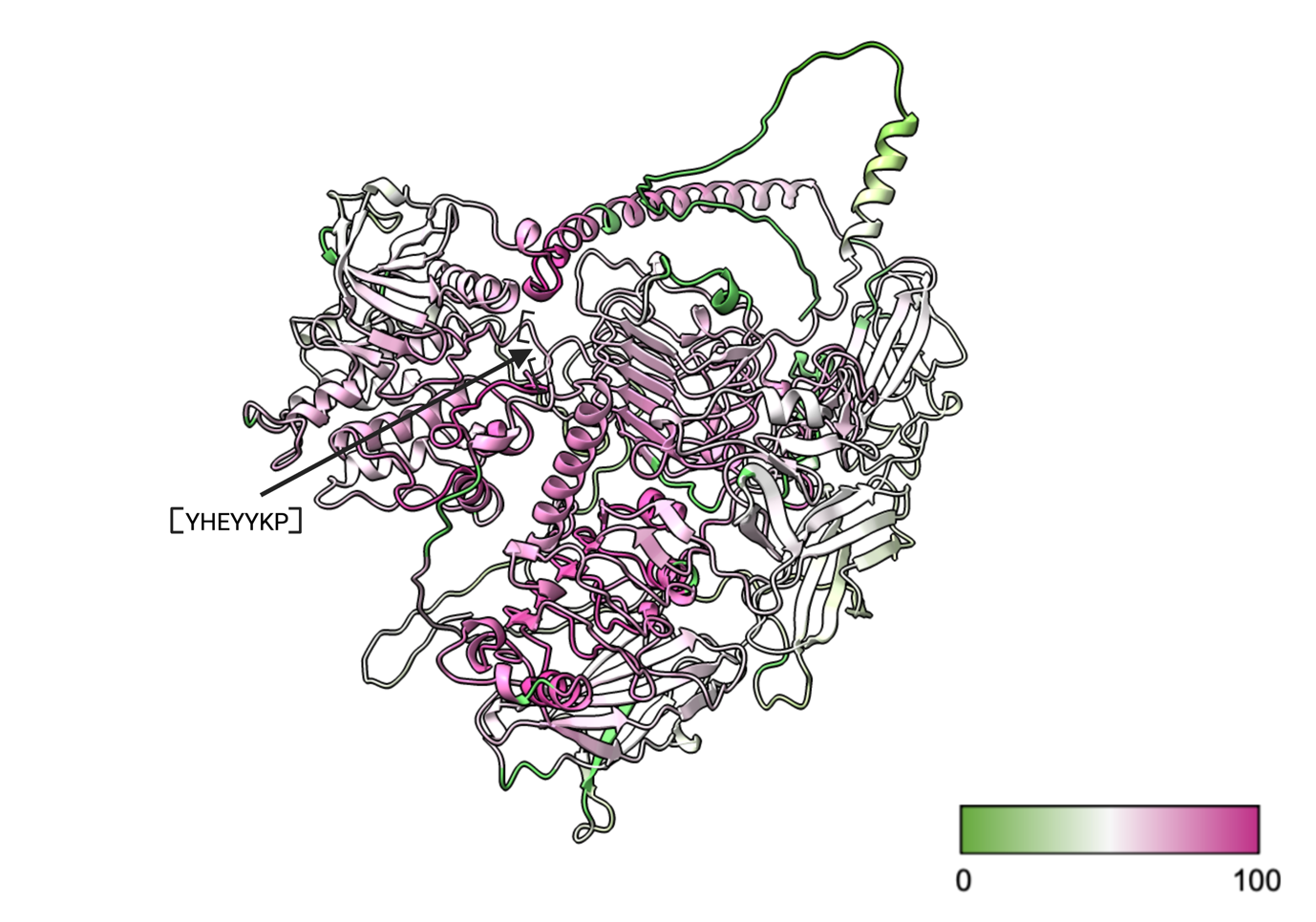
 P06213 (*Homo sapiens*) vs HCON_00066590 (*Haemonchus contortus*)


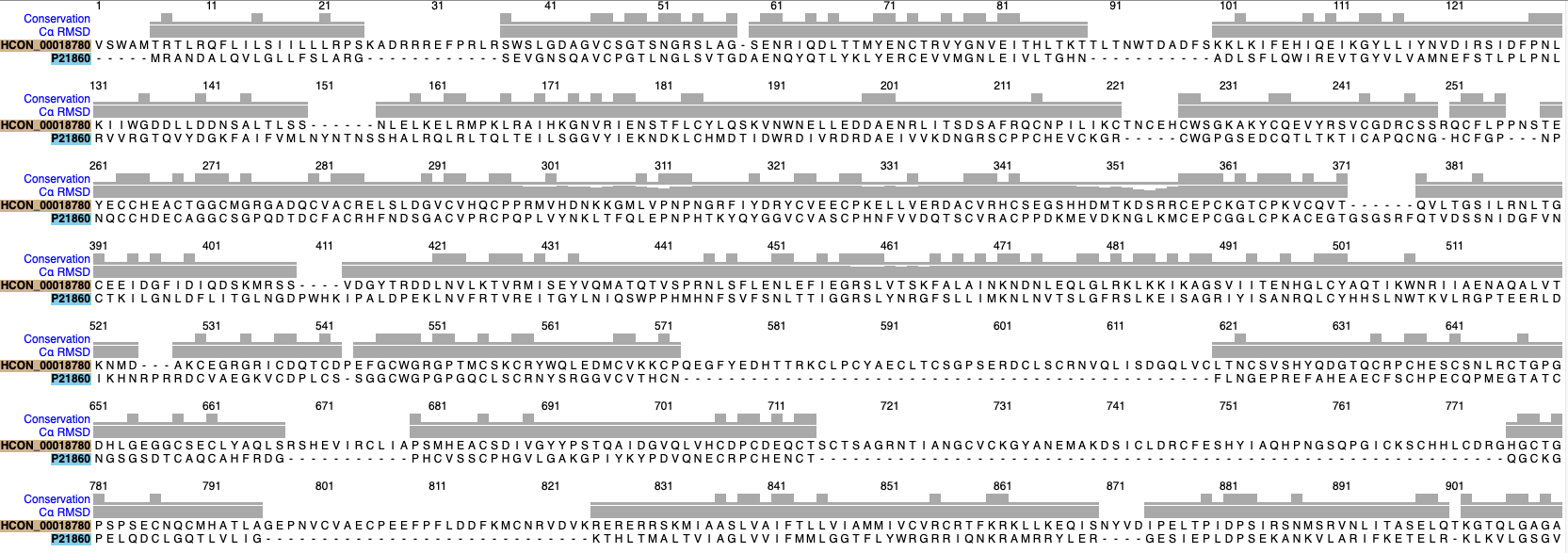


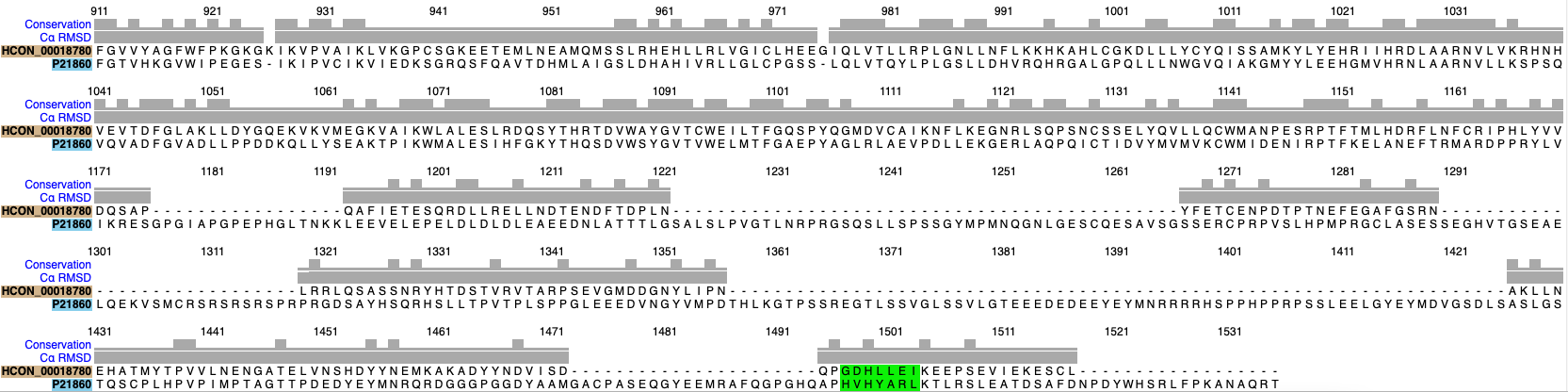


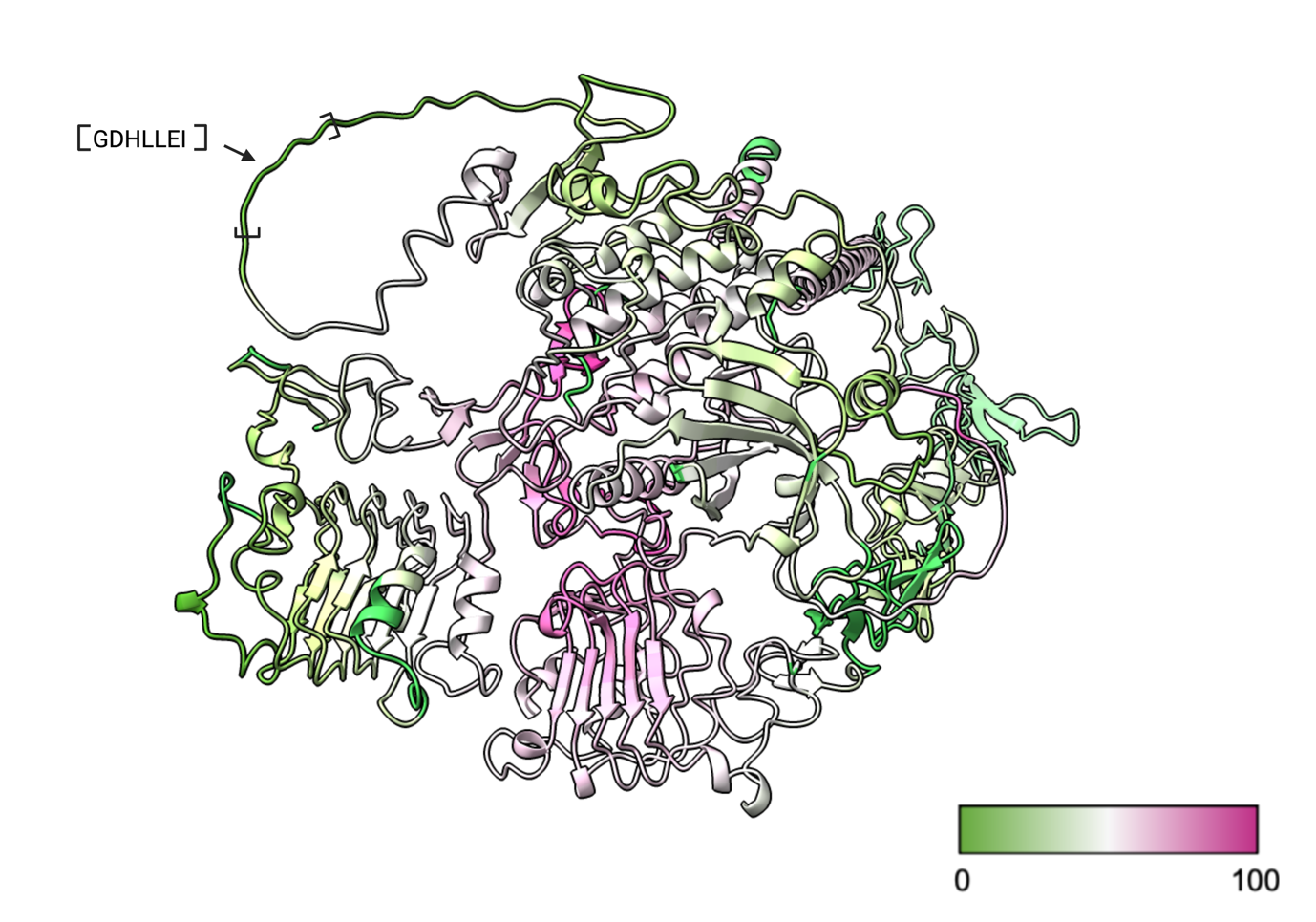


P21860 (*Homo sapiens*) vs HCON_00018780 (*Haemonchus contortus*)


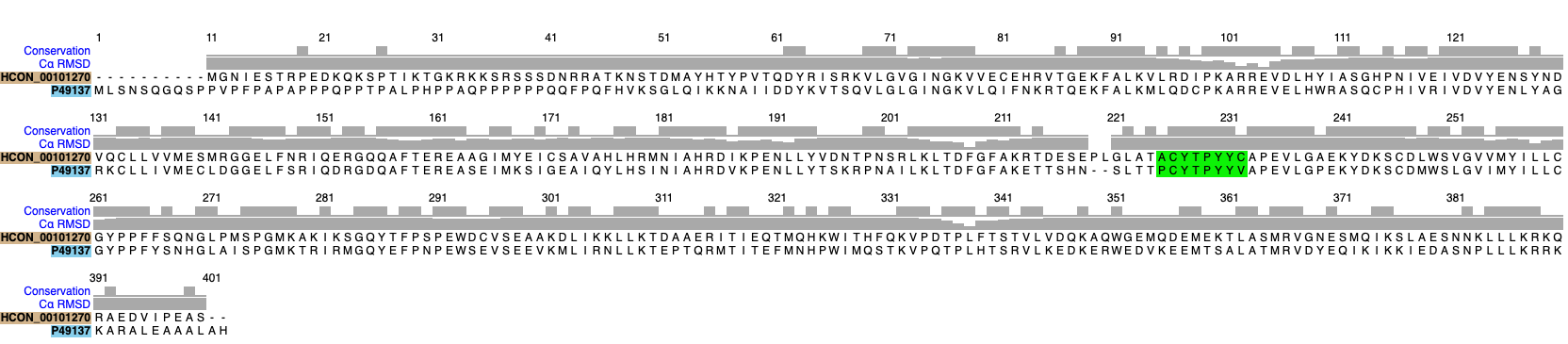


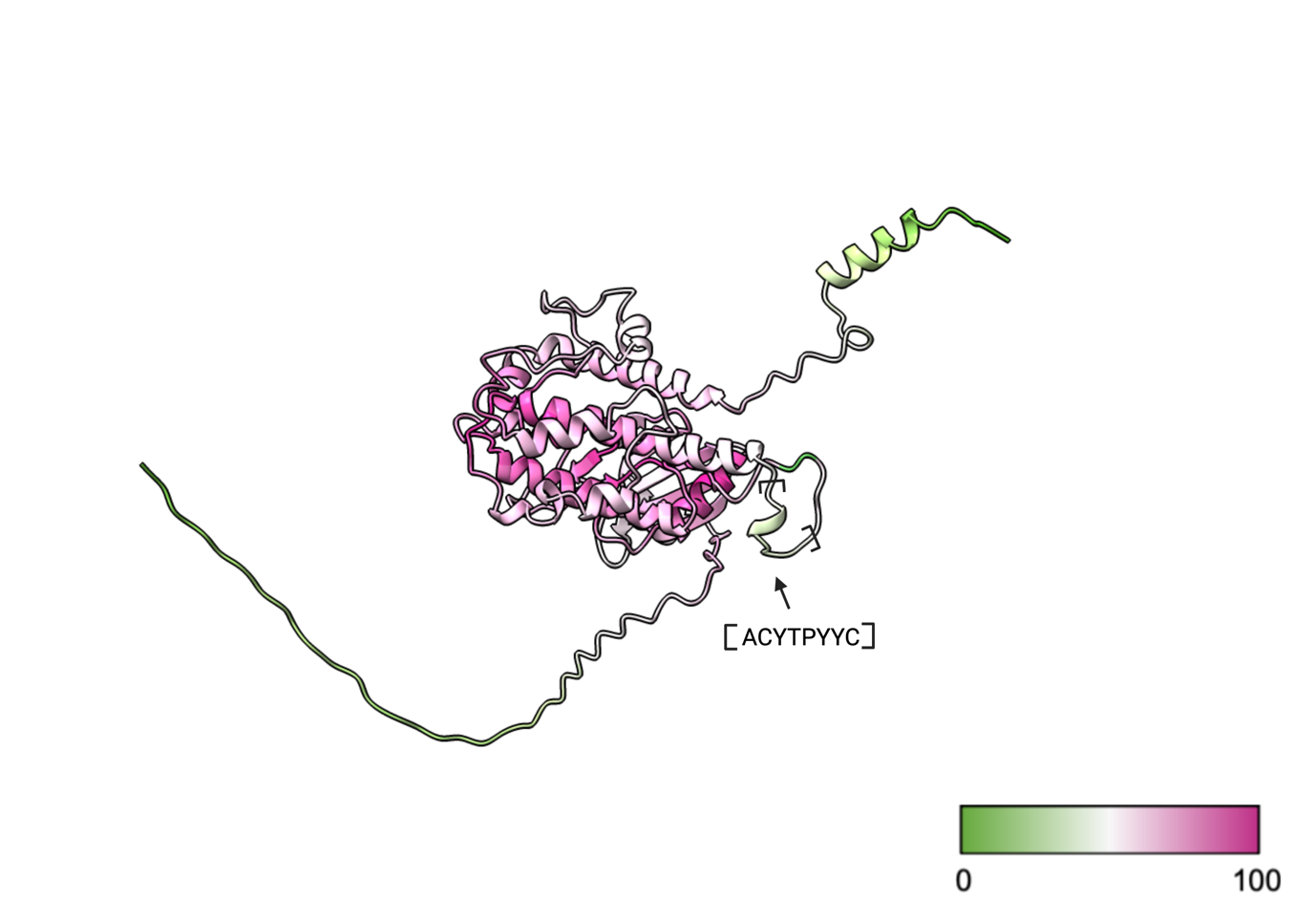


P49137 (*Homo sapiens*) vs HCON_00101270 (*Haemonchus contortus*)


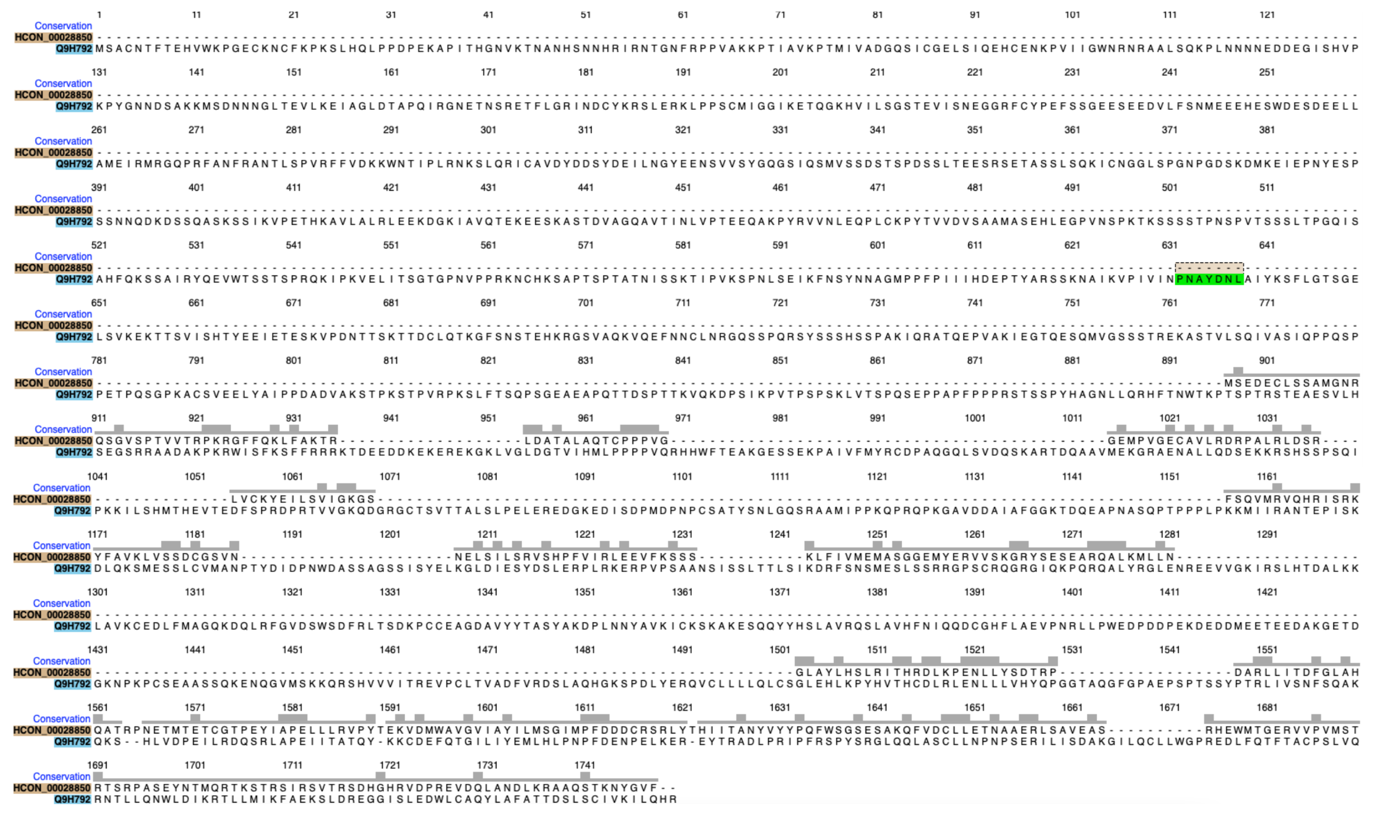


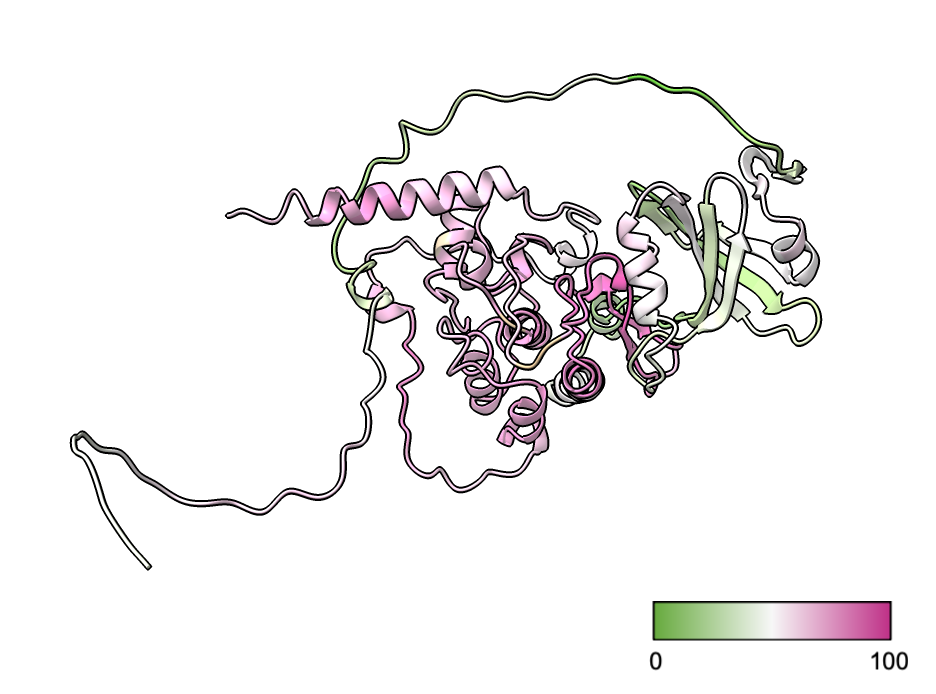


Q9H792 (*Homo sapiens*) vs HCON_00028850 (*Haemonchus contortus*)
